# Pediatric Drug Adherence and Parental Attention: Evidence From Comprehensive Claims Data

**DOI:** 10.1002/hec.70062

**Published:** 2025-11-15

**Authors:** Josh Feng, Matthew J. Higgins, Elena Patel

**Affiliations:** ^1^ The University of Utah Salt Lake City Utah USA; ^2^ Tulane University New Orleans Louisiana USA; ^3^ Max Planck Institute for Innovation and Competition Munich Germany; ^4^ National Bureau of Economic Research Cambridge Massachusetts USA

**Keywords:** COVID‐19, drug adherence, parental attention, pediatric health

## Abstract

Using comprehensive U.S. drug claims data, we show that adherence to asthma control medication declined during the COVID‐19 pandemic. We find that young children exhibited a 40 percent decrease in adherence by the end of 2020. The responses were less negative for older children and positive for adults. We provide additional evidence that parental attention played a role in driving this decrease, based on heterogeneity by pre‐pandemic mail order usage and number of parental scripts. Policy implications for improving pediatric adherence are discussed.

## 1 Introduction

Poor drug adherence increases the likelihood of negative clinical outcomes and increases the cost of healthcare (e.g., Osterberg and Blaschke 2015). Although several studies have examined determinants of non‐adherence for adults—highlighting factors such as insurance coverage and socioeconomic status—there is limited systematic evidence on the drivers of *pediatric* adherence. Yet, the need for such evidence is substantial because the mechanisms that influence adherence for children are likely to differ, because they are: (i) generally eligible for insurance; and (ii) rely on caregivers for filling and administering medication.

Control medication for asthma is an important leading case of low pediatric drug adherence.^1^ As noted in Currie (2009), asthma is the most common chronic health condition among children and also the leading driver of emergency room (ER) visits and hospitalizations among children.^2^ Asthma can generally be controlled via daily control medication, but children only take medication on about 30–40% of days, on average (Asamoah‐Boaheng et al. 2021). Schlender et al. (2012) project that increased adherence to control medication can reduce ER visits and hospitalizations by at least 50%. More broadly, poor drug adherence among pediatric asthma patients has been shown to negatively affect long‐term health, educational attainment, and income (e.g., Miller et al. 2009; Currie 2009).

Our study sheds light on pediatric adherence to asthma control medication by analyzing comprehensive prescription claims data in the context of the COVID‐19 pandemic. First, we establish that pediatric adherence to asthma control medication declined significantly during the pandemic, with the largest impact on the youngest children.^3^ Second, we find that age and pre‐pandemic use of mail‐order pharmacies significantly moderate the effect of the pandemic, even after accounting for air quality, school closures, socioeconomic factors, and other COVID‐related measures. Finally, we use data from a nationally representative survey to provide further suggestive evidence that parental attention plays a role in driving the observed adherence patterns.

We construct our research design around the start of the COVID‐19 pandemic for two main reasons. First, the pandemic created a broad and unprecedented shock, making it inherently important to measure its impact on adherence to asthma medication, a major contributor to pediatric health outcomes. Second, decomposing the response can provide insights into the mechanisms driving the low levels of pediatric adherence during non‐pandemic times, while also potentially offering lessons for how adherence may respond to other large‐scale disruptions such as macroeconomic downturns.

Our primary analysis relies on a proprietary transactions database that captures near‐population‐level prescription drug claims from the U.S. market. The richness of this database conveys several advantages over previous studies, which typically use small sample surveys, self‐reported information, and track individuals only to the extent that they remain covered by the same employer. First, these data are large enough to permit a fine‐grained subsample analysis to investigate the mechanisms underlying our result. Second, these data allow us to track individuals over time, regardless of insurer. Finally, the transactional nature of the data makes it more reliable than self‐reported survey evidence for measuring an important health behavior.

Using this dataset, we build a patient‐by‐month panel of monthly medication coverage rates for roughly 2 million pediatric asthma patients who filled prescriptions in the 2018 to 2020 period. Crucially, we focus on patients who take “control” medication, which is necessary for managing the disease and preventing asthma attacks. We compare monthly adherence outcomes in 2020 to those from 2018 to 2019, both overall and within individuals, to estimate the response to the pandemic. Overall, we find a sustained decrease in pediatric drug adherence of 13% during the pandemic. However, the dynamics of this effect are more sharply negative later in 2020, especially for the youngest children in our sample. By December 2020, preschool‐aged children experienced a 40% decrease in adherence rates, while adherence among school‐aged children and teenagers fell by 25% and 21% over the same period, respectively.

The fact that the youngest group exhibited the largest negative effects may be consistent with several explanations. One possibility is that very young children are less able to communicate their needs and therefore may be more dependent on parental attention (Fitzpatrick et al. 2009).^4^ Another is that parents may have perceived a lower need for asthma management because reduced daycare attendance and exposure to other viruses can lead to fewer acute events.^5^ This distinction matters for the effectiveness of potential policies such as automatic refills.

To probe these mechanisms, we first estimate a horse‐race event that includes factors affecting perceived need (e.g., local school closures and changes in air quality), measures of access (e.g., telehealth laws, pre‐pandemic usage of mail order, and insurance), socioeconomic characteristics. Most factors are not predictive of the *change* in adherence at the individual level, with two exceptions: age and pre‐pandemic use of mail order. The latter is associated with a counteracting effect equal to 40% of the average decline in adherence.

We also present evidence pointing to parental attention as an important driver of the observed response. Because our IQVIA data does not contain family identifiers, we turn to a complementary data source, the Medical Expenditure Panel Survey (MEPS), to show that the declines in adherence are much smaller when parents also pick up their own prescriptions. In addition, we present suggestive evidence that decreased adherence translated into worse health outcomes in the youngest children.

We contribute to the literature on the determinants and consequences of drug adherence. This literature has noted several sources of poor adherence, including cognitive impairment, poor patient‐provider relationships, side effects, lack of insurance, and high out‐of‐pocket costs (Chandra et al. 2010; A. Finkelstein et al. 2012; Brot‐Goldberg et al. 2017; Huskamp et al. 2003; Bosworth et al. 2011; Osterberg and Blaschke 2015; McQuaid and Landier 2018). In addition, this literature has documented several consequences of poor adherence, including worse clinical outcomes and increased risk of hospitalization and death (e.g., Chandra et al. 2010; Osterberg and Blaschke 2015). Our evidence speaks to an understudied and influential factor related to pediatric adherence: parental attention. Our findings on the importance of parental attention are consistent with survey evidence (Matsui 2007). The size of our estimated response within children is on par with, if not larger than, the responses to large changes in insurance parameters estimated in the literature (Chandra et al. 2010; A. Finkelstein et al. 2012; Brot‐Goldberg et al. 2017).

We also contribute to a nascent literature studying the impact of the pandemic on prescription drug adherence (Kaye et al. 2020; Ferraro et al. 2021; Yang et al. 2022; Haapanen et al. 2022; Clement et al. 2021). We are the first to document longer‐run negative effects on pediatric drug adherence using large‐scale administrative data and a measure of adherence that is based on coverage rates. By comparison, the existing literature generally finds a positive relation based on small sample studies and more subjective adherence measures.

Our findings also contribute to the literature on pediatric health and the role of parents. The literature has documented a strong relationship between pediatric health and parental socioeconomic status (SES), one that strengthens as children grow older (Case et al. 2002; Currie and Stabile 2003; Conti et al. 2010). Yi et al. (2015) provides evidence of the reallocation of resources by parents in response to pediatric health shocks. Focusing on adults, Fadlon and Heien Nielsen (2019) provides quasi‐experimental evidence of within‐family spillovers, including drug adherence. Our results suggest that differences across children in their parents' attention to chronic disease management could be a driver of the increasingly steep health‐SES gradient as children age.

Finally, we contribute to the large body of work documenting the effects of macroeconomic conditions on health behaviors and outcomes. Ruhm (2000) documents the pro‐cyclical nature of mortality; subsequent work has examined mortality differences by age (Dehejia and Lleras‐Muney 2004; Van Den Berg et al. 2006; Miller et al. 2009) and employment (Sullivan and Wachter 2009). Burgard and Hawkins (2014) focuses specifically on the Great Recession, finding decreases in healthcare use, especially among African Americans and Hispanics, and among those individuals with less education. We expand this literature by studying pediatric adherence during COVID, which suggests that the youngest children could be negatively affected during other downturns.

## 2 Background

### 2.1 Pediatric Asthma

Asthma is one of the most common chronic conditions affecting children. In 2018, 8.5 million (11.6%) children under 18 reported ever having been diagnosed with asthma, and 5.5 million (7.5%) children reported still having asthma.^6^ Asthma can be managed by taking short‐term “as‐needed” medications to help relieve symptoms during an asthma attack and long‐term “control” medications to help prevent attacks and control symptoms by reducing airway inflammation and preventing the narrowing of airways (Karaca‐Mandic et al. 2012).^7^ In 2018, 72% of young children with asthma reported using a prescription inhaler within the last 3 months, and 55% of young children with asthma reported taking preventative medication.

Adherence to prescriptions is a key issue, both for the management of chronic disease generally and specifically among asthmatic individuals. According to a 2003 World Health Organization (WHO) report, “…increasing the effectiveness of adherence interventions may have a far greater impact on the health of a population than any improvement in specific medical treatment…” (Sabate and World Health Organization 2003). Poor adherence has been shown to directly affect disease management, including increased risk of hospitalization and death (Osterberg and Blaschke 2015; Asamoah‐Boaheng et al. 2021). The costs and health consequences of mismanaged asthma can be significant. When well controlled, asthma rarely leads to hospitalization, but non‐adherence increases the odds of experiencing an asthma attack and emergency room visits (Weiss et al. 1992).

In our study, we focus on adherence to asthma control medication in our analysis. First, adherence measures are less appropriate for as‐needed medication, which is only used during an asthma attack. As a result, these prescriptions may be filled infrequently and used with significant lag relative to their fill date, making fill patterns difficult to interpret in the context of adherence measures. Moreover, declines in as‐needed fills may also capture changes in the incidence of asthma attacks (e.g., from improved air quality) rather than from changes in adherence behavior.^8^ In contrast, control medications are intended for daily use, making adherence measures derived from fill and refill data more appropriate and interpretable.

As noted earlier, pediatric adherence to asthma is quite low on average, and the medical literature has noted several potential drivers related to caregivers. A review of studies by Asamoah‐Boaheng et al. (2021) finds average proportion of days covered (PDC), a commonly used adherence measure, ranging from 30 to 40%. Some of this is driven by low refill rates, which Fitzpatrick et al. (2009) measure to be 38%. The same study summarizes several possible drivers of low adherence and refill rates, mostly focusing on the role of the caregiver. These include younger children not being able to “express subtle changes in respiratory symptoms to their caregivers”, incorrect beliefs on the part of caregivers in terms of when medication is needed, and “family stress” that makes it difficult to incorporate prescription fills and office visits “into family routines.”

### 2.2 Key COVID‐19 Events

The WHO declared COVID a pandemic on March 11, 2020, followed by a U.S. declaration of a national emergency on March 13, 2020. Six days later, California became the first state to issue a stay‐at‐home order; other states quickly followed. In what follows, we outline several significant events that are likely to affect drug adherence.

Several significant pieces of federal legislation were passed in response to the onset of the pandemic. First, the Coronavirus Preparedness and Response Supplemental Appropriations Act, enacted on March 6, 2020, included a waiver allowing Medicare providers to offer telehealth services. In addition, Federal officials encouraged states and insurers to provide similar flexibility under private insurance, which many did (Volk et al. 2021).^9^ Second, the Families First Coronavirus Response Act, enacted on March 18, 2020, mandated that states could not disenroll any beneficiary who had Medicaid coverage through the end of 2020. Around this time, many states also increased the quantity limits on prescriptions, typically from 30 to 90 days, and relaxed limits on early refills. Third, the Coronavirus Aid Relief and Economic Security Act (CARES), enacted on March 27, 2020, provided $2 trillion worth of emergency assistance for individuals, families, and businesses. For example, CARES provisioned up to $1200 in individual Economic Impact Payments beginning April 15, 2020. Collectively, these three pieces of legislation likely expanded access to care, increased quantities of drugs per claim, and made the time between claims more irregular.

School closures and re‐openings were also highly relevant events, especially in the context of pediatric adherence, because schools often play a key role in administering treatment routines for children. After the national emergency declaration, all public schools in the U.S. were closed for in‐person learning. These closures were recommended or mandated for the rest of the 2019–2020 academic year in most states. During the start of the 2020–2021 academic year, there was additional heterogeneity in mandated in‐person learning.^10^

## 3 Data and Methodology

### 3.1 Data

Our primary data source is the IQVIA Longitudinal Prescription Claims (LRx) dataset. Each entry in these data corresponds to a drug claim and includes anonymized patient identifiers, prescriber zip code, drug identifiers, fill date, days supplied, method of fill, and primary payer. We provide detailed variable descriptions for these Supporting Information S1 data, including sources, in Supporting Information S1: Table A2. These data cover the near‐universe of prescription drug claims in the U.S.

We study an extract of these data that captures all prescriptions associated with the treatment of asthma. The detail in these data permits us to generate a continuous, monthly adherence measure by combining information about fill and refill dates and days of medication supplied to each individual. We describe this measure in detail below. The scope of these data not only allows us to derive representative results for the population but also allows us to precisely estimate sub‐population effects.

We supplement these data with local population data based on the prescriber's zip code, including race, income, education, occupation, population density, and access to health insurance. We also characterize the development of the COVID pandemic using measures of school closures, air quality, and access to telehealth using several external sources. These data and sources are described in Supporting Information S1: Table A3.

Because IQVIA does not contain information on parents or caregivers, we incorporate two Supporting Information S1 datasets. First, we use data from the nationally representative Medical Expenditure Panel Survey (MEPS), which helps us measure parental socio‐economic status and other family‐specific contextual factors, such as the number of parental prescriptions. A major disadvantage of MEPS, however, is the sample size. This survey only tracks about fifteen thousand individuals over the 2019–2020 period and, as a result, captures very few pediatric patients taking asthma medication. We also use older data from Truven Health's MarketScan database, which tracks both families and dates of prescriptions, to provide more general statistics on how often children and parents simultaneously fill prescriptions.

### 3.2 Measuring Drug Adherence

We construct a monthly, patient‐level panel data set for patient cohorts in 2018–2020. Cohorts are defined based on individuals who filled at least one control medication in January–March of a given year.^11^ We then follow these individuals from March through December of that year to measure adherence outcomes. January and February fills are used only to establish cohort membership and initial stock levels, but these months are not included as outcome months. Because cohorts are constructed separately for each year, the same individual may appear in multiple cohorts if they filled prescriptions in multiple years. Patients are assigned a unique identifier in the LRx database, which allows us to track patients across cohorts.

For each patient month, we compute the share of days covered based on the stock of medication on hand and the flow of prescription fills and refills: stock decreases by one each day from the earliest observed transaction in the LRx database and is replenished with each new prescription. We aggregate this measure to obtain the proportion of days covered (PDC) by control medication for each patient‐month. We interpret year‐over‐year changes in monthly coverage rates as changes in drug adherence. Our measure is similar to other continuous measures commonly used for assessing adherence in asthma pharmacy claims databases (Lam and Fresco 2015; Asamoah‐Boaheng et al. 2021). Our final analysis sample contains roughly 2 million children under the age of 18 per cohort. Finally, we characterize each patient‐year based on the characteristics of the first prescription filled during the first 3 months of the year. For example, we note the payer type for a given individual and whether they fill prescriptions via mail order versus at a retail pharmacy.

### 3.3 Prep‐COVID Pediatric Drug Adherence

**FIGURE 1:**
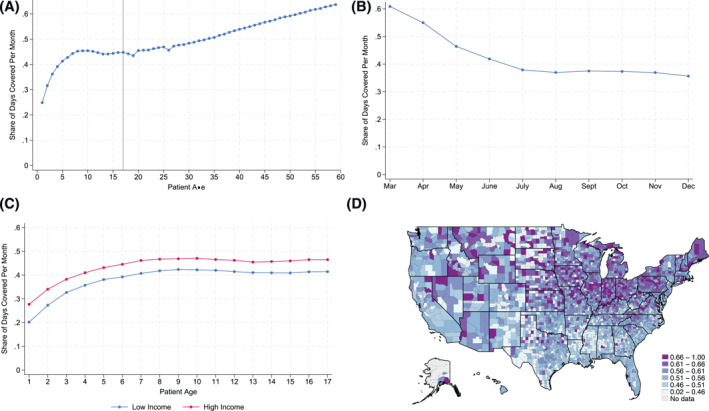
Pre‐pandemic coverage rates. (A) Average monthly coverage rate. (B) Monthly pediatric coverage rate. (C) Pediatric coverage rate, by income. (D) Pediatric coverage rate. The four panels in Figure 1 plot the average monthly coverage rates in 2018 and 2019 for asthma patients taking control medication. All statistics reflect coverage rates between March and December, which defines our analysis window. Panel (A) depicts average coverage rates by age. Panel (B) depicts monthly pediatric coverage rates. Panel (C) depicts monthly pediatric coverage rates for children with a provider located in a high‐ and low‐income zip code. Panel (D) depicts average pediatric coverage rates by county, based on the provider's zip code.

Figure 1, Panel (A) depicts the average monthly coverage rate for users younger than 60 years old. The average PDC measure ranges from less than 0.3 for the youngest patients to over 0.5 for adults over 40.^12^ These values are within the range of PDC values found in the literature (Asamoah‐Boaheng et al. 2021).^13^

Panel (B) depicts monthly patterns in pediatric coverage for our sample. Because we require that at least one prescription be filled between January and March, PDC rates are highest in the first 3 months of the year.^14^ PDC rates gradually fall across the first 6 months of the year, stabilizing at roughly 35% by the end of the year. In Panel (C), we decompose pediatric coverage rates by income, showing that coverage rates are lower for patients who live in low‐income geographies. This is consistent with the fact that children living in low‐income areas are more likely to be asthmatic and that income is a negative predictor of pediatric adherence (Matsui 2007). Finally, Panel (D) depicts the average pediatric coverage rate by county in 2019, revealing the scope of geographic variation that exists across the U.S.

Additionally, Table 1 reports baseline statistics describing our pediatric analysis sample in 2019 (Col 1), and then separately across three age subgroups (Cols 2–4). The first subgroup roughly corresponds to children who are preschool‐aged, the second to children in elementary and middle school, and the third to children in high school.^15^ We observe 1.9 million unique users, of which 24, 50, and 26% are under 6, aged 6 to 12, and aged 13 to 17, respectively. Panel (A) describes individual characteristics of patients, while Panel (B) describes geographic characteristics based on their providers.

**TABLE 1:** Sample statistics: Pediatric asthma patients, January–March 2019.

	Under 18 (1)	Under 6 (2)	6 to 12 (3)	13 to 17 (4)
Panel A: Individual characteristics				
Adherence rate	0.56	0.51	0.58	0.58
*>* 30% Days covered	0.66	0.62	0.68	0.67
Patient age	9.18	3.41	8.96	14.83
Share female	0.42	0.40	0.41	0.45
Medicaid payer	0.13	0.13	0.13	0.13
Third party payer	0.86	0.86	0.85	0.86
Panel B: 2018 local geographic characteristics				
Per‐capita income, 2018	31,905	31,755	31,897	32,057
Minority share of population, 2018	0.26	0.26	0.26	0.25
Share population with some college, 2018	0.21	0.21	0.21	0.21
Share of population in urban area	0.89	0.90	0.89	0.89
Medicaid expansion state	0.56	0.54	0.55	0.57
Observations	1,925,675	458,648	962,432	504,595

*Note:* This table provides summary statistics describing the population of pediatric asthma prescription users in 2019. Adherence statistics reflect prescriptions filled between January and March, and individual characteristics reflect the first prescription filled in 2019. Local geographic characteristics are matched based on the zip code of the provider associated with the first prescription filled. Columns 2–4 report summary statistics for subpopulations based on age. Variable definitions provided in Supporting Information S1: Appendix Tables A2 and A3.

### 3.4 Methodology

We estimate the effect of the COVID pandemic on drug consumption by comparing coverage rates in each month of 2020 to coverage rates the same month in 2019:

(1) $$y_{imt}=\beta_{0}+\beta_{1}Y\;2020+\beta_{2}Y\;2018+\gamma_{z}+u_{imt}$$

Here, *y*_*imt*_ measures the monthly drug coverage (PDC) for individual *i* in month *m* in year *t*, and *β*_1_ estimates any change in drug consumption in 2020 compared to 2019. In addition, *β*_2_ estimates any change in drug consumption from 2018 to 2019. We interpret *β*_1_ as a test of the effect of the pandemic on drug consumption. *β*_2_ provides a placebo test related to whether trends in year‐over‐year drug consumption existed prior to the pandemic. In some specifications, we include a provider zip‐code fixed effect, *γ*_*z*_, based on the prescriber of the first prescription filled for a given cohort, and in other specifications, we include individual fixed effects.

For specifications that include a provider zip‐code effect, $\beta_{1}$ is identified by comparing PDC rates within prescriber locations across the same month in 2019 and 2020. This approach absorbs time‐invariant characteristics of the local prescribing environment and patient mix at the zip‐code level. When we instead include individual fixed effects, the comparison is within the same patient across years, for a given month. This specification imposes stronger continuity requirements by restricting the analysis to patients who appear in our sample across multiple years, mechanically emphasizing more chronic and persistent users of prescription drugs. By comparison, the zip‐code fixed effect specification only requires that zip codes have ongoing prescribing activity to be included. In both cases, the identifying variation comes from within‐unit changes in PDC in 2020, relative to the 2019 baseline. All estimates are clustered at the state level.

In addition to the above model, we also estimate specifications that drop the *Y* 2018 variable, so that estimates of *β* ^ˆ^_1_ reflect the difference in any given month of 2020 compared to average consumption in 2019 and 2018. We take advantage of this simplification to conduct empirical tests of the relative importance of patient and geographic characteristics, *π*_*i*_, in explaining *β* ^ˆ^_1_:

(2) $$y_{imt}=\beta_{0}+\beta_{1}Y\;2020+\chi \pi_{i}+\psi \pi_{i}\times Y\;2020+\gamma_{z}+u_{imt}$$

For example, suppose *π*_*i*_ is a dummy variable equal to one for patients who filled their first prescription of the year by mail. Then *ψ* captures the estimate of the pandemic on monthly coverage rates for patients who fill their prescriptions by mail relative to those who use alternative delivery channels, or the relative effect of the pandemic.

Table 2 reports summary statistics from 2018, 2019, and 2020, measured based on prescriptions filled in the first three months of each year. Panel (A) describes the characteristics of patients and patient consumption, and Panel (B) describes the geographic characteristics associated with the zip code of their provider. Patient characteristics appear to be balanced across all three cohorts included in our analysis. We interpret the imbalance in patient consumption in 2018 compared to 2019 and 2020 as mechanically due to the way we build our consumption data, which is calculated based on prescription fills and refills that we observe beginning in January 2018. This leads to a “burn‐in” issue for 2018, because we do not observe scripts from late 2017.

**TABLE 2:** Balance: Pediatric asthma patients, January–March.

	2018	2019	2020
Panel A: Individual characteristics			
Adherence rate	0.47	0.56	0.56
*>* 30% Days covered	0.58	0.66	0.66
Patient age	9.13	9.18	9.34
Share female	0.42	0.42	0.42
Medicaid payer	0.13	0.13	0.12
Third party payer	0.85	0.86	0.87
Panel B: 2018 local geographic characteristics			
Per‐capita income, 2018	32,076.38	31,904.98	32,117.20
Minority share of population, 2018	0.26	0.26	0.26
Share population with some college, 2018	0.21	0.21	0.21
Share of population in urban area	0.90	0.89	0.90
Medicaid expansion state	0.56	0.56	0.56
Observations	2,092,890	1,925,675	2,039,886

*Note:* This table provides summary statistics describing the population of pediatric continuing asthma prescription users based on their January‐March 2018, 2019, and 2020 prescriptions. Variable definitions provided in Supporting Information S1: Appendix Tables A2 and A3.

Table 3 presents statistics related to the mechanics of prescriptions in each year in our sample. The average number of scripts per year at the patient level is about 5, and the average days supplied is about 35 days. About half of the scripts are marked as refills. The fractions of scripts that involve cash payments (1%–2%) and mail delivery (2%) are very low in the pediatric sample. All statistics are similar across years, except the number of scripts filled (lower) and days supplied per script (higher) in 2020.

**TABLE 3:** Annual patient‐level summary statistics: Pediatric asthma prescriptions.

	2018	2019	2020
Number Rx	5.51	5.35	4.92
Avg days supplied per Rx	34.84	35.52	37.09
Share Rx, refill	0.46	0.47	0.48
Share Rx, medicaid	0.13	0.13	0.12
Share Rx, third party	0.85	0.86	0.87
Share Rx, cash	0.02	0.01	0.01
Share Rx, mail delivery	0.02	0.02	0.02
Annual days supplied	185.99	182.39	174.52
Observations	2,092,890	1,925,675	2,039,886

*Note:* This table provides summary statistics describing annual asthma prescriptions for pediatric users in our analysis in 2018, 2019, and 2020. Variable definitions provided in Supporting Information S1: Appendix Tables A2 and A3.

The key identification assumption behind our study design is that year‐over‐year variation in monthly drug consumption would have been similar in 2020 compared to 2019 if not for the onset of the pandemic. We revisit this assumption and present evidence in support of this identification strategy after presenting our core results in Section 4.

## 4 Empirical Findings

### 4.1 Impact of COVID on Adherence

**FIGURE 2:**
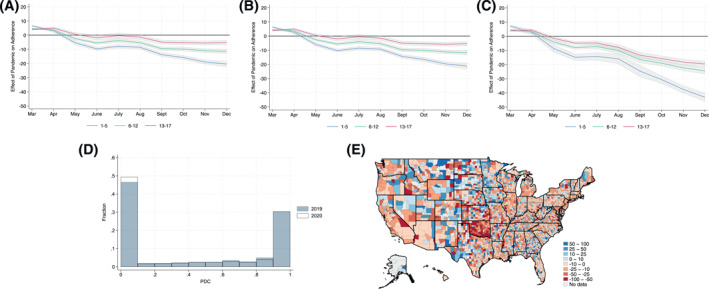
Effect of the pandemic on monthly pediatric drug adherence. (A) Mean adherence. (B) Provider zipcode fixed effects. (C) Individual fixed effects. (D) PDC: 2019 versus 2020 (E) Effect by county: Aug–Dec. Figure 2, Panels (A)–(C) plot the monthly difference in pediatric drug adherence from 2019 to 2020 by age of the patient based on Equation (1). All results are scaled by the 2019 average pediatric adherence rate. Panel (A) depicts mean changes in drug adherence. Panel (B) includes state fixed effects. Panel (C) includes individual fixed effects. Panel (D) plots the distribution of the PDC measure at the patient‐month level for March to December of 2019 and 2020. Panel (E) plots the estimated mean effect of the pandemic for patients aged 1 to 17 within county averaged across August–December; data ranges reflect the following parts of the distribution: 0–10, 10–25, 25–50, 50–75, 75–90, 90–100.

Figure 2, Panel (A) depicts the estimated mean monthly change in pediatric adherence using our core specification without any fixed effects. Estimates, scaled by average monthly consumption in 2019, are reported separately for preschool‐aged children (aged 1–5), school‐aged children (aged 6–12), and teenagers (aged 13–17).^16^

We find substantial heterogeneity in the effect of the pandemic from March through December. For March, we find that adherence rates increased by 5%–8% across all pediatric age groups, likely reflecting stockpiling with the onset of the pandemic. Pediatric adherence responses became negative starting in June and fell even further in the fall and through the end of 2020. Furthermore, these estimates show that the youngest group exhibited the largest effects. Overall, we find that preschool‐aged children experienced a 20% decrease from their expected coverage rate in December 2020, whereas school‐aged children and teenagers experienced decreases of 10 and 5%, respectively.

Figure 2, Panels (B) and (C), present results that incorporate zip code and individual fixed effects. We find similar qualitative patterns throughout, with larger quantitative effects when including individual fixed effects: preschool‐aged children experienced a 40% decrease in December 2020, whereas school‐aged children and teenagers experienced decreases of 25 and 22%, respectively.^17^ Ultimately, we estimate that adherence fell by an average of 13% across all children from March through December when incorporating individual fixed effects.^18^

Figure 2, Panel (D) presents an alternative way to view our estimates. Specifically, we plot the distribution of PDC in 2019 (gray boxes) and 2020 (white, outlined boxes). We see a systematic reduction in PDC across the entire distribution, and especially for patient‐months with 0%–10% share of days covered. This is consistent with the decrease in adherence that we measure using a variety of econometric specifications, as previously described.

We also verify that response sizes are similar in percentage terms when we use other measures of adherence. As noted above, our main measure of adherence is PDC. Asamoah‐Boaheng et al. (2021) note that evidence in the health literature suggests that having a PDC below 50% is associated with negative long‐term health outcomes. Therefore, we re‐estimate Equation (1) using indicators for whether an individual's PDC was above 50% and above 30%. We also re‐run our analysis using Medication Possession Ratio, another commonly used adherence measure as the outcome.^19^ Figure A1 shows that the percentage response is similar when using these alternate adherence measures.

Next, we report the within‐county scaled effects of the pandemic in Panel (E). Nearly all counties saw a decrease in pediatric coverage rates, regardless of underlying differences in population, political landscape, access to health care and health insurance, localized evolution of the pandemic, and many other factors, underscoring both the severity and the scope of the COVID response. We revisit these and other COVID‐specific factors in greater detail below.

**FIGURE 3:**
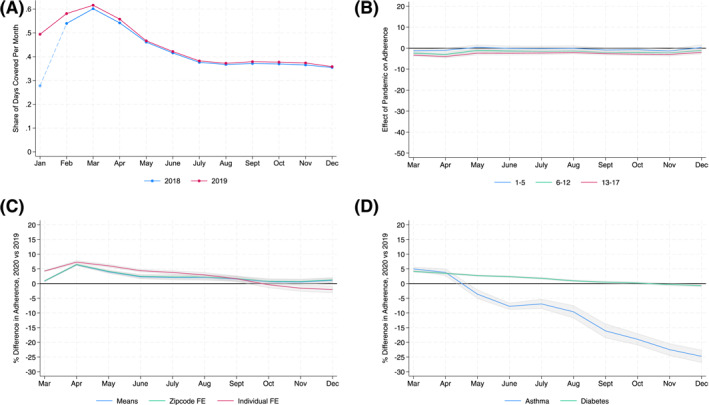
Identification and data validation. (A) 2018 and 2019 coverage rates. (B) 2018 and 2019 differences. (C) Adherence effect: Adults 18 to 59. (D) Other disease: Pediatric diabetes. Panel (A) plots the average coverage rates for pediatric users in our analysis sample in 2018 and 2019. Panel (B) plots differences in coverage rates in 2018 compared to 2019 for pediatric patients. Panel (C) plots differences in coverage rates in 2020 compared to 2019—or the effect of the pandemic; results are scaled by the average pediatric adherence rate each month in 2019. Panel (D) plots the effect of the pandemic on adherence rates for pediatric asthma patients together with pediatric diabetes patients using an individual fixed effect specification.

Finally, Figure 3 presents several results related to our identification assumption. First, Panel (A) shows that pediatric adherence rates were very similar for March through December in 2018 and 2019, providing evidence of a stable control sample.^20^ Panel (B) provides detailed comparisons within pediatric age groups, again showing very similar rates in 2018 and 2019. Figure 3 also provides additional contextual results. Panel (C) presents estimates of the effect of the pandemic on adult adherence under different fixed effects specifications, showing that adults *increased* adherence throughout 2020; this rules out mechanisms that would have similar effects on both adults and children. Panel (D) compares results for asthma to diabetes and finds that pediatric diabetes patients exhibited some stockpiling but no negative adherence response in 2020. We revisit this in our discussion below on mechanisms.

### 4.2 Horserace Analysis

Our core results, including the heterogeneity by age groups, could be driven by various mechanisms related to the ones discussed in Section 2.1. One explanation consistent with the data is that the youngest children are most sensitive to parental attention because they are unable to communicate symptoms or provide reminders. Another interpretation is that parents perceive a decrease in need because of reduced symptoms due to improved air quality or daycare attendance, consistent with the findings in Farber et al. (2003). The distinction matters for some potential policies. For example, sending reminders or automatic refills would be beneficial if parents are forgetting to fill prescriptions; however, it would be less useful if parents do not perceive a need.

To assess the potential contribution of these factors, we run a horserace regression based on Equation (2) that tests for mediating effects. We include interactions of the 2020 effect with factors that may drive perceived need (school closings, air quality, and COVID cases), factors related to access (insurance coverage, mail order, and telehealth laws), age, and other socioeconomic measures. Table 4 reports estimates for individuals under 18, providing estimates for all months, March to July, and August to December. The first three columns focus on the average response, as captured by the “2020” variable. Differences in these estimates highlight the early stockpiling and subsequent negative effects (−10pp in the August to December period vs. −5.78pp overall).

**TABLE 4:** Collective impact of mechanisms: Horserace.

	Mar–Dec (1)	Mar–July (2)	Aug–Dec (3)	Mar–Dec (4)	Mar–July (5)	Aug–Dec (6)
2020	−0.0578***	−0.0154***	−0.100***	−0.0292***	−0.0139*	−0.0445***
	(0.00236)	(0.00210)	(0.00306)	(0.00762)	(0.00609)	(0.0105)
Age × 2020				0.00219*** (0.000144)	0.00128*** (0.000130)	0.00311*** (0.000192)
Mail × 2020				0.0362*** (0.00254)	0.0331*** (0.00248)	0.0394*** (0.00352)
AQI improvement × 2020				0.00111	0.00198	0.000232
				(0.00234)	(0.00255)	(0.00255)
High income × 2020				−0.00530**	−0.00192	−0.00869***
				(0.00183)	(0.00166)	(0.00229)
High education × 2020				−0.00785*** (0.00169)	−0.00572*** (0.00141)	−0.00997*** (0.00255)
White collar × 2020				−0.00795	−0.00237	−0.0135
				(0.00517)	(0.00367)	(0.00788)
High minority × 2020				0.000556	−0.000886	0.00200
				(0.00177)	(0.00178)	(0.00221)
Medicaid payer × 2020				0.0115**	0.0102**	0.0128
				(0.00428)	(0.00323)	(0.00660)
Medicaid expansion × 2020				0.00680*	0.00667*	0.00693
				(0.00321)	(0.00307)	(0.00413)
Telehealth × 2020				−0.00127	−0.00270	0.000171
				(0.00400)	(0.00308)	(0.00590)
Urban × 2020				0.0125*** (0.00267)	0.0126*** (0.00242)	0.0125*** (0.00345)
High school closure × 2020				0.0113*** (0.00268)	0.0120*** (0.00244)	0.0105** (0.00345)
Control mean	0.431	0.489	0.372	0.431	0.489	0.372
Individual FE	x	x	x	x	x	x
Observations	60,584,510	30,292,255	30,292,255	60,584,510	30,292,255	30,292,255

*Note:* Estimates are based on a regression model that compares adherence in 2020 to adherence in 2019 and 2018, following Equation (1). Columns 1 and 3 report the average effect across all months, and columns 2, 3, 5, and 6 report estimates for a subset of months as indicated by column headers. Control means reflect average pediatric adherence in the designated months in 2019. Variable definitions provided in Supporting Information S1: Appendix Table A2 and A3. All specifications include individual fixed effects. Standard errors are clustered at the state level.

∗, ∗∗ and ∗∗∗ denote 5%, 1%, and 0.1% significance levels, respectively.

**FIGURE 4:**
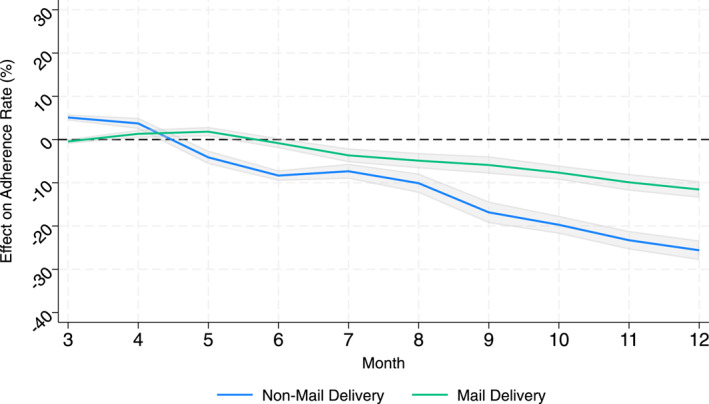
Heterogeneity by delivery channel. Figure 4 plots the effect of the pandemic on pediatric adherence by the delivery channel used by the individual before the onset of the pandemic.

The next three columns of Table 4 repeat this analysis but include a large set of interaction terms to assess potential mediating factors. We find that the quantitatively large and statistically significant mediating factors for August to December are age and pre‐pandemic usage of mail‐order pharmacies. The estimates in Column 6 of Table 4 suggest that a 16‐year increase in *Age* offsets half of the average effect for August through December (−10pp from Column 3).^21^ For mail order, the mediating effect is 40% of the average effect in the August to December period (3.94pp in column 6 vs. −10pp average in Column 3). We also provide graphical evidence in Figure 4, focusing just on the differences between users of mail order before the pandemic and other individuals. Consistent with the horse‐race estimates, we see less stockpiling for individuals using mail delivery, followed by less negative responses later in the year.

For the remaining factors, we find minimal quantitative significance. Local school closure rates do not meaningfully mediate the average effect in the latter months of 2020 (+1pp effect on a baseline of −10pp). This is consistent with the longitudinal pattern in our core estimates, namely that the negative pediatric response becomes stronger even as schools reopen in the fall. We also find quantitatively small effects for access variables, including telehealth laws (precisely zero). In addition, we find that air quality has a limited mediating impact (precisely zero).^22^ Our regression also includes interactions with other county and state‐level demographics, socioeconomic measures, and population health statistics. Overall, we find minimal evidence that these factors had quantitatively large mediating effects.^23^ We provide more detailed results and discussion in Supporting Information S1: Appendix C.

#### 4.2.1 Discussion of Horserace Evidence

The horserace estimates indicate that age and pre‐pandemic usage of mail order are strongly correlated with an individual's adherence response in 2020. The age gradient may reflect either changes in perceived need, due to reductions in other respiratory diseases that affect younger children (e.g., RSV) or lower daycare attendance, or changes in parental attention.

By contrast, the mail‐order result aligns more closely with the parental attention mechanism. Mail order offers logistical advantages over retail pharmacies: (1) refills, while not automatic, can be ordered online; (2) prescriptions can cover longer periods due to regulator differences.^24^ These factors reduce reliance on routine parental effort and may buffer against disruptions in attention or family routines. If perceived need were the sole driver of reduced adherence, mail‐order use would not be expected to exert such a strong mitigating effect, since it still requires an active parental decision.

### 4.3 Additional Evidence Related to Parental Attention

Here, we provide additional evidence related to the parental attention channel, using data sources that contain family identifiers (MEPS, MarketScan) and additional IQVIA data we collected on prescriptions for diabetes medication.

Our analysis of MEPS proceeds in two steps. First, we replicate our core finding that monthly coverage rates decrease for children. To do so, we create an individual‐by‐year panel for users of asthma medication in 2019 who continue to be surveyed by MEPS in 2020.^25^ We compute the total days supplied for each individual and year.^26^ We then estimate the average change in adherence rates between 2019 and 2020 for children from different age groups using a Poisson regression *Y*_*it*_ *∼ Poisson*(*λ*_*it*_):

(3) $$\lambda_{\text{it}}=\alpha_{i}+\beta \;\cdot \;I_{t=2020}+\epsilon_{\text{it}}$$

where *i* indexes the individual and *t* the year (2019 or 2020). The primary outcome of interest is the total number of days of medication filled. Table 5, Panel (A) reports these results. We find that the average quantity of medication decreased for all children, with larger effects for younger age groups. In contrast, adults exhibit no effect.

**TABLE 5:** Evidence on adherence and parents from MEPS.

	Total number of medication days covered (Q)				
	All (1)	Under 5 (2)	6–12 (3)	13–17 (4)	Adults (5)
Panel A: Average response by age group					
2020	−0.846***	−1.222***	−0.897***	−0.457	−0.101
	(0.162)	(0.343)	(0.237)	(0.248)	(0.0530)
Person FE	x	x	x	x	x
No. individuals	318	91	124	103	1353

Panel B: Horserace, all kids		
	Asthma Q	Non‐asthma Q
	(1)	(2)
2020	−1.005***	−0.462***
Log Rx × 2020	(0.153) 0.205*	(0.0845) 0.157**
	(0.0992)	(0.0589)
Δ Mental × 2020	−0.110	−0.0312
	(0.386)	(0.137)
Lost job × 2020	0.826**	−0.236
	(0.270)	(0.253)
Lost ins × 2020	−0.391	−0.289
	(0.839)	(0.294)
Education × 2020	−0.103*	−0.0168
	(0.0439)	(0.0316)
Log hourly wage × 2020	0.0294	−0.0491
	(0.0987)	(0.0621)
Person FE	x	x
No. Individuals	318	566

*Note:* Estimates from fixed‐effects Poisson regressions for Equations (3) and (4). All log variables refer to log(1+variable). “Rx” refers to the number of distinct scripts recorded in MEPS and “Q” refers to the today number of days supplied. In Panel (B), the models denote the parental measure we are using in the interaction term. All measures are demeaned to preserve the interpretation of the overall effect. “Rx” refers to the total adult scripts in 2020. ΔMental refers to the average change in self‐reported mental health status across all adults in the family, with higher values representing worse status. Education refers to the higher number of years of education of any adult in the family. “Lost Ins” and “Lost Job” indicate whether any adult in the family lost insurance coverage or lost employment across the three rounds of the survey in 2020, respectively. “Log Wage” refers to the log of the sum of hourly wage across all adults in the family during the first round in 2020. Standard errors are clustered at the individual level.

* *p <* 0.05.

** *p <* 0.01.

*** *p <* 0.001.

Second, we construct data describing the parents of children who take asthma medication to study parent‐level mediating factors. For each asthmatic child in 2019, we construct a set of parental measures based on MEPS. These measures include (1) log of one plus the total number of prescriptions for parents in 2020 (Log *Rx*), (2) the change in self‐reported mental health state from 2019 to 2020 averaged across parents (Δ*Mental*), (3) whether any adult lost employment in 2020 (*Lost Job*), (4) whether any adult in the family lost health insurance coverage in 2020 (*Lost Ins*), (5) the highest education level of any parent (*Education*), and (6) hourly wages (Log *Wage*).

Formally, we estimate the following Poisson specification:

(4) $$\lambda_{it}=\alpha_{i}+\beta I_{t=2020}+\sum_{j}\gamma_{j}I_{t=2020}\left(X_{i}^{j}-{\bar{X}}^{j}\right)+\varepsilon_{it}$$

where $X_{i}^{j}$ are the factors discussed above and ${\bar{X}}^{j}$ are the population means. Results are reported in Table 5, Panel B, Model 1.

We find that parental usage of medication is a significant predictor of the response size. The offsetting effect of log adult prescriptions is quantitatively large and statistically significant. An increase from zero to the median number of adult prescriptions is associated with 58% of the baseline effect from Panel (A). We also find a large positive estimate for parental job loss, but this effect is not present in the subsequent non‐asthma analysis. Importantly, other factors, such as parental education and income, have quantitatively smaller correlations with response size. Panel (B), Model 2 repeats the horse‐race analysis for the most common non‐asthma medication taken by children (primarily allergy and attention medication). We again find an average decrease in adherence and find similar mediating effects for parental prescriptions. This suggests that the patterns found in asthma apply at least to other chronic medications.

Next, we provide evidence on the plausibility of the parental attention channel by documenting the timing of prescription fills within families. To do so, we use claims data from MarketScan.^27^ In the last year of our sample, we find that a given pediatric asthma prescription is picked up on the same day as any parent prescription 13.2% of the time and that a given pediatric asthma prescription is picked up within 9 days of a parent's prescription 50% of the time. Supporting Information S1: Table A10 provides additional statistics. We note that the presence of the mail order channel could color the interpretation of these statistics, but mail order is generally a much less used channel versus retail pharmacies, even in adults.

These co‐occurrence rates confirm the plausibility of the parental attention channel. As shown earlier, adult adherence rates increased in 2020. If the co‐occurrence rates were close to 100%, this would make it implausible that the decrease in pediatric prescriptions is driven by adults forgetting. Furthermore, assuming that 13.2% of pediatric prescriptions are unaffected by changes in parental attention because of co‐occurrence, the remaining 86.8% of prescriptions would only have to be missed 34% of the time to generate the negative 30% response we observe for the youngest children in December 2020, a reasonable rate.

Finally, we also provide additional evidence using adherence data for pediatric diabetes. Unlike asthma, non‐adherence to insulin for children with Type 1 diabetes would generate immediate feedback. If we still see a decline in adherence, it could suggest something other than parental attention is driving decreases in both disease classes. To provide evidence, we apply the same methodology to insulin scripts from IQVIA. Figure 3, Panel (D) compares the scaled effect of the pandemic on adherence for pediatric diabetes and asthma patients. For diabetes, adherence is essentially unchanged, apart from some initial stockpiling, in stark contrast to our finding of a persistent decline in adherence for asthma. We note that this test does not rule out changes in perceived demand as an explanatory channel for the asthma results.

### 4.4 Evidence on Health Impacts

As noted earlier, the medical literature suggests that reduced adherence to control medication will lead to worse long‐run health outcomes. However, identifying such effects is difficult for several reasons. First, our IQVIA data is not linked to medical claims, which precludes a comparison of health outcomes across individuals with differing adherence responses. Second, Binney et al. (2024) find that asthma hospitalizations had been significantly decreasing leading up to the pandemic, with half as many hospitalizations in 2019 versus 2012. The authors attribute this to better management of asthma.

Despite these issues, we use MEPS data to provide some evidence on health trends among pediatric asthma patients.^28^ For each age group (1–5, 6–12, 13–17, and adults) and year between 2013 and 2022, we calculate the average number of emergency room visits plus hospital stays among asthma patients.^29^ We find patterns that are generally consistent with non‐adherence having subsequent negative health impacts. Consistent with Binney et al. (2024), Figure A4 shows a decreasing trend in the number of events leading up to 2020 and a sharp drop in 2020. In 2021 and 2022, we only find a consistent increase within the youngest age group, consistent with non‐adherence being a key issue among the youngest asthma patients in the latter half of 2020. Supporting Information S1: Table A11 provides formal estimates at the individual level that suggest a large relative increase in hospitalization rates for the youngest children after 2020. However, the pre‐trends and limited sample size in MEPS make it difficult to address the issues with great precision. Again, we note that this evidence does not rule out changes in perceived demand as a driver of the decreased adherence.

## 5 Conclusion

Using large‐scale transaction data, we have documented a significant decrease in pediatric adherence to asthma control medication during the COVID‐19 pandemic, from an already low baseline. We find the largest negative effects in the youngest children and provide suggestive evidence that it led to worse health outcomes in that group.

We also use data on enrollment in mail order and parental fills that parental attention may be a key factor in driving the observed decrease, something behavioral interventions could mitigate. These include reminders, automatic mail delivery, and other forms of assistance. The impact of these interventions might be especially impactful during times of individual and aggregate macroeconomic turmoil ‐ for example, following a job loss or during recessions.

Overall, our findings underscore the critical role that families play in managing chronic pediatric health conditions. Moreover, our results draw attention to heterogeneity in adherence across disease classes. Children with conditions that provide immediate feedback, such as diabetes, appear less vulnerable. On the other hand, our work suggests that the management of pediatric asthma is intertwined with the demands on parental attention that are increased by the pandemic. This contrast may speak to variation in the sensitivity of other pediatric health behaviors to parental attention.

## Conflicts of Interest

The authors declare no conflicts of interest.

## Supporting information


Supporting Information S1

## Data Availability

The data that support the findings of this study are available from IQVIA. Restrictions apply to the availability of these data, which were used under license for this study. Data are available from the author(s) with the permission of IQVIA.

## Endnotes

^1^
Control medication is intended to be taken daily to manage asthma, as opposed to “as‐needed” medication, which is used in the case of an acute asthma attack.

^2^
According to the National Health Interview Survey (NHIS), one in five children in the U.S. has been diagnosed with asthma.

^3^
Our primary measure of adherence is based on the proportion of days covered (PDC) in each month.

^4^
Pandemic‐era survey evidence from parents with children under age 6, the group showing the biggest negative response, suggest high levels of emotional distress and burn out that worsened over time (Rapid Survey Project 2022).

^5^
Farber et al. (2003) provide interview evidence that parents commonly view control medication as “symptom relieving” rather than preventative, even though the medically recommended regime is daily usage.

^6^
Summary statistics are based on the author's calculations using the 2018 NHIS.

^7^
Supporting Information S1: Table A1 provides detailed classifications for asthma medication in our sample, based on the classifications in Karaca‐Mandic et al. (2012) and Sayed et al. (2022).

^8^
Excluding as‐needed medication also helps us avoid complications related to the Albuterol shortage in the first months of the pandemic (Hendeles and Prabhakaran 2020).

^9^
Centers for Medicare and Medicaid Services, FAQs on Availability and Usage of Telehealth Services Through Private Health Insurance Coverage in Response to Coronavirus Disease 2019 (CMS, March 24, 2020).

^10^
Detailed statistics provided by Education Week are available at https://www.edweek.org/ew/section/multimedia/map‐covid‐19‐schools‐open‐closed.html.

^11^
Patients appear in multiple cohorts if they have filled a control medication in January‐March in multiple years.

^12^
We exclude patients described as 0 years old when filling their first control medication prescription in January ‐ March of a given year. For this reason, the youngest patient in our sample is 1 year old.

^13^
Table S9 of Asamoah‐Boaheng et al. (2021) summarizes studies in the literature that measure PDC. The studies range from 0.32 to 0.41 in average PDC, with lower average PDC values for children than for adults.

^14^
However, the average rates are not going to be close to one, because a fill on the 31st of a month will only count as a 3.22% adherence rate if there is a gap in medication coverage before the fill.

^15^
We characterize patient age based on the age associated with the first prescription filled in the calendar year to avoid within‐year attrition.

^16^
in Supporting Information S1: Table A4 reports corresponding unscaled coefficients.

^17^
Supporting Information S1: Table A5 reports corresponding unscaled coefficients for within‐zip code and within‐patient specifications.

^18^
This number is based on Column (1) of Table 4. Although control medication is intended for daily use, individuals may ration medication during the initial stockpiling period, so that usage differs from our PDC measure. Pooling across the whole year helps us reliably estimate an overall effect.

^19^
Medical Possession Ratio is measured based on days filled in a given month divided by total days.

^20^
The January and February adherence rates in 2018 are lower than in 2019, partly reflecting the “burn‐in” issue noted earlier.

^21^
Supporting Information S1: Figure A2 reports non‐parametric estimates of the effect by age to show that linearity assumptions are not driving our results. In the August to December period, the estimates are monotonic by age, with minimal effects for 17‐year‐olds and a large negative response for the youngest children. Finally, we find quantitatively negligible age effects within the adult population (Supporting Information S1: Table A7).

^22^
In unreported results, we do find significant mediating effects of AQI for users of “as‐needed” medication.

^23^
Surprisingly, traditional measures of high SES (high income and education) seem to be associated with negative point estimates, indicating a larger decrease in adherence in these areas. We stress that our results do not mean these factors do not matter more broadly for adherence, because we do observe higher adherence rates in higher income areas in 2018 and 2019.

^24^
In our data, the average mail order script contains a 60‐day supply of medication versus 30 days for prescriptions filled at retail pharmacies.

^25^
Although MEPS contains rounds of surveys within a given year, the dates of the prescriptions are not available, so a monthly analysis is not possible.

^26^
Supporting Information S1: Appendix D.1 provides information about data, empirical design, and summary statistics.

^27^
We have access to data from 1996 to 2013. Supporting Information S1: Appendix D.2 provides detailed methodology and results.

^28^
Supporting Information S1: Appendix D.3 provides details on methodology.

^29^
2022 is the latest year for which MEPS has posted data. Other commonly used datasets like the one used in Binney et al. (2024) are also only available through 2022.
