# Supplementary material for: Pediatric Drug Adherence and Parental Attention: Evidence From Comprehensive Claims Data
Source: Health Econ. 2025 Nov 15;35(2):346–59. doi: 10.1002/hec.70062 (PMC12779210; doi:10.1002/hec.70062)
Supplement: Supplementary file 1 — Supporting Information S1 [file HEC-35-346-s001.docx]

**APPENDIX: “Pediatric Drug Adherence and Parental Attention:**

**Evidence from Comprehensive Claims Data”**

1. **Drug Classifications and Variable Definitions**

Table A1: Drug Classification Crosswalk: LRx Database

| **Marketed Product Name** | **Total Pediatric Scripts** | **USC Code** | **Classification** |
| --- | --- | --- | --- |
| MONTELUKAST SODIUM | 23,832,218 | 28500 | maintenance |
| ALBUTEROL SULFATE | 14,673,416 | 28112 | acute |
| FLOVENT HFA | 8,843,200 | 28410 | maintenance |
| PROAIR HFA | 8,784,009 | 28111 | acute |
| ALBUTEROL SULFATE HFA | 8,544,319 | 28111 | acute |
| VENTOLIN HFA | 6,068,265 | 28111 | acute |
| BUDESONIDE | 2,925,650 | 28410 | maintenance |
| QVAR REDIHALER | 2,050,127 | 28410 | maintenance |
| SYMBICORT | 1,437,621 | 28431 | maintenance |
| ADVAIR HFA | 885,086 | 28431 | maintenance |
| PROVENTIL HFA | 815,239 | 28111 | acute |
| QVAR | 653,260 | 28410 | maintenance |
| DULERA | 625,478 | 28431 | maintenance |
| PROAIR RESPICLICK | 457,569 | 28111 | acute |
| PULMICORT | 419,619 | 28410 | maintenance |
| ADVAIR DISKUS | 395,680 | 28431 | maintenance |
| LEVALBUTEROL HCL | 360,092 | 28112 | acute |
| LEVALBUTEROL TARTRATE HFA | 271,257 | 28111 | acute |
| ASMANEX HFA | 266,074 | 28410 | maintenance |
| ARNUITY ELLIPTA | 257,637 | 28410 | maintenance |
| PULMICORT FLEXHALER | 211,642 | 28410 | maintenance |
| FLUTICASONE PROPIONATE/SA | 177,138 | 28431 | maintenance |
| BUDESONIDE/FORMOTEROL FUM | 171,385 | 28431 | maintenance |
| BREO ELLIPTA | 164,509 | 28431 | maintenance |
| ASMANEX TWISTHALER 30 MET | 148,084 | 28410 | maintenance |
| DUPIXENT | 137,160 | 86230 | maintenance |
| WIXELA INHUB | 108,949 | 28431 | maintenance |
| XOLAIR | 107,488 | 28900 | maintenance |
| FLOVENT DISKUS | 101,412 | 28410 | maintenance |
| SPIRIVA RESPIMAT | 97,160 | 28121 | maintenance |
| ALVESCO | 46,356 | 28410 | maintenance |
| ASMANEX TWISTHALER 60 MET | 45,445 | 28410 | maintenance |
| XOPENEX HFA | 38,031 | 28111 | acute |
| LEVALBUTEROL HYDROCHLORID | 37,732 | 28112 | acute |
| ASMANEX TWISTHALER 120 ME | 14,622 | 28410 | maintenance |
| SINGULAIR | 13,621 | 28500 | maintenance |
| TRELEGY ELLIPTA | 12,810 | 28432 | maintenance |
| ZAFIRLUKAST | 12,431 | 28500 | maintenance |
| NUCALA | 10,494 | 28900 | maintenance |
| THEOPHYLLINE ER | 9,068 | 28131 | maintenance |
| CROMOLYN SODIUM | 8,040 | 28210 | maintenance |
| SEREVENT DISKUS | 5,299 | 28118 | maintenance |
| XOPENEX | 5,122 | 28112 | acute |
| FASENRA | 4,427 | 28900 | maintenance |
| TERBUTALINE SULFATE | 4,181 | 28113 | maintenance |
| THEO-24 | 4,138 | 28131 | maintenance |
| AEROSPAN | 3,212 | 28410 | maintenance |
| LEVALBUTEROL | 3,142 | 28112 | acute |
| THEOPHYLLINE | 1,882 | 28131 | maintenance |
| ZILEUTON ER | 700 | 28500 | maintenance |
| FASENRA PEN | 656 | 28900 | maintenance |
| ALBUTEROL SULFATE ER | 556 | 28113 | acute |
| ASMANEX TWISTHALER 14 MET | 520 | 28410 | maintenance |
| AIRDUO RESPICLICK 113/14 | 480 | 28431 | maintenance |
| PROAIR DIGIHALER | 389 | 28111 | acute |
| AIRDUO RESPICLICK 232/14 | 214 | 28431 | maintenance |
| AIRDUO RESPICLICK 55/14 | 174 | 28431 | maintenance |

Table A2: Variable Definitions: IQVIA LRx Database

| **Variable** | **Definition** |
| --- | --- |
| Drug Adherence | Share of days in a given month covered by the oldest, unused prescription. Constructed based on the following Rx specific information: the date the prescription was filled and the number of days that the prescription is intended to last, as instructed by the healthcare provider  *Source: LRx, IQVIA*. |
| Age | The age of the patient based on the first prescription filled in a given  calendar year. *Source: LRx, IQVIA* |
| Gender | Gender of Patient (M,F) *Source: LRx, IQVIA* |
| Provider Zip Code | The zipcode for the provider’s primary address based on the first prescription filled in a given calendar year. *Source: LRx, IQVIA*. |
| Payer | Primary Method of Payment: Cash, Medicaid, Third Party, Medicare,  Medicare Part D based on the first prescription filled in a given calendar year. Note: Managed Medicaid is categorized as a Third Party Payer *Source: LRx, IQVIA* |
| Mail Order | Dummy variable equal to one if the pharmacy distribution channel was  indicated to be “Mail” based on the first prescription filled in a given calendar year. *Source: LRx, IQVIA* |

Table A3: Variable Definitions: Supplemental Data Sources

| **Variable** | **Definition** |
| --- | --- |
| Medicaid Expansion | Dummy variable equal to one for patients with a provider located in  a Medicaid Expansion State as of 12/31/2019 *Source: Kaiser Family Foundation* |
| High Minority Population | Dummy variable equal to one for patients with a provider located in  a county in the top 25th percentile of the distribution of the share of the population that is non-white. *Source: Torch Insights, drawn from the American Community Survey* |
| High Education | Dummy variable equal to one for patients with a provider located in a  county in the top 25th percentile of the distribution of the share of the population that has at least some college experience. *Source: Torch Insights, drawn from the American Community Survey* |
| High Income | Dummy variable equal to one for patients with a provider located in  a county in the top 25th percentile of the distribution of per-capita income. *Source: Torch Insights, drawn from the American Community Survey* |
| Urban | Dummy variable equal to one for patients with a provider located in  a zip code where more than 75% of the population is categorized as  living in an urban area. *Source: U.S. Census* |
| High School Closure | Dummy variable equal to one for patients with a provider located in a  county that is in the top 25th percentile when ranked based on the share of schools with at least a 50% drop in school attendance in September 2020 compared to September 2019. *Source: U.S. School Closure and Distance Learning Database, Parolin and Lee (2021)* |
| AQI Drop | Dummy variable equal to one for patients with a provider located in  a county that experienced a decrease in the average Air Quality Index (AQI) compared with 2018 to 2019 trends, which reflects an *improvement* in air quality *Source: Environmental Protection Agency* |
| Telehealth | Dummy variable equal to one for patients with a provider located in  a state that required insurers to cover telemedicine services *Source: Commonwealth Fund Issue Brief* |

Table A4: Mean Effect of Pandemic on Adherence: Variation By Age

| Mar | Apr | May | Jun | July | Aug | Sept | Oct | Nov | Dec | Obs |
| --- | --- | --- | --- | --- | --- | --- | --- | --- | --- | --- |
| (1) | (2) | (3) | (4) | (5) | (6) | (7) | (8) | (9) | (10) |  |
| *Panel A: 1–5 years old* |  |  |  |  |  |  |  |  |  |  |
| *Y* 2018 -0.00684^∗∗∗^ | -0.00493 | 0.00161 | -0.000236 | -0.000514 | -0.000127 | -0.00269 | -0.00231 | -0.00423^∗^ | 0.000958 |  |
| (0.00188) | (0.00265) | (0.00253) | (0.00214) | (0.00201) | (0.00195) | (0.00176) | (0.00172) | (0.00176) | (0.00222) |  |
| *Y* 2020 0.0372^∗∗∗^ | 0.0148^∗∗∗^ | -0.0214^∗∗∗^ | -0.0339^∗∗∗^ | -0.0242^∗∗∗^ | -0.0252^∗∗∗^ | -0.0417^∗∗∗^ | -0.0492^∗∗∗^ | -0.0601^∗∗∗^ | -0.0629^∗∗∗^ |  |
| (0.00154) | (0.00288) | (0.00302) | (0.00233) | (0.00220) | (0.00204) | (0.00230) | (0.00241) | (0.00254) | (0.00329) |  |
| Control Mean / N 0.565 | 0.495 | 0.397 | 0.350 | 0.308 | 0.293 | 0.302 | 0.311 | 0.317 | 0.307 | 1,419,607 |
| *Panel B: 6–12 years old* |  |  |  |  |  |  |  |  |  |  |
| *Y* 2018 -0.0151^∗∗∗^ | -0.0171^∗∗∗^ | -0.00531^∗^ | -0.00609^∗∗^ | -0.00637^∗∗^ | -0.00555^∗∗^ | -0.00845^∗∗∗^ | -0.00824^∗∗∗^ | -0.00885^∗∗∗^ | -0.00420^∗^ | |
| (0.00184) | (0.00185) | (0.00220) | (0.00213) | (0.00223) | (0.00180) | (0.00162) | (0.00183) | (0.00180) | (0.00204) | |
| *Y* 2020 0.0293^∗∗∗^ | 0.0209^∗∗∗^ | -0.0121^∗∗∗^ | -0.0245^∗∗∗^ | -0.0154^∗∗∗^ | -0.0208^∗∗∗^ | -0.0385^∗∗∗^ | -0.0395^∗∗∗^ | -0.0434^∗∗∗^ | -0.0435^∗∗∗^ | |
| (0.00150) | (0.00238) | (0.00247) | (0.00249) | (0.00315) | (0.00272) | (0.00271) | (0.00306) | (0.00286) | (0.00319) | |
| Control Mean / N 0.631 | 0.576 | 0.489 | 0.444 | 0.403 | 0.395 | 0.404 | 0.400 | 0.395 | 0.379 3,038,615 | |
| *Panel C: 13–17 years old* |  |  |  |  |  |  |  |  |  |  |
| *Y* 2018 -0.0213^∗∗∗^ | -0.0231^∗∗∗^ | -0.0117^∗∗∗^ | -0.0109^∗∗∗^ | -0.00939^∗∗∗^ | -0.00834^∗∗∗^ | -0.0106^∗∗∗^ | -0.0114^∗∗∗^ | -0.0115^∗∗∗^ | -0.00737^∗∗∗^ |  |
| (0.00183) | (0.00219) | (0.00227) | (0.00208) | (0.00189) | (0.00156) | (0.00147) | (0.00152) | (0.00167) | (0.00165) |  |
| *Y* 2020 0.0248^∗∗∗^ | 0.0293^∗∗∗^ | 0.00272 | -0.00832^∗^ | -0.00146 | -0.00558 | -0.0197^∗∗∗^ | -0.0207^∗∗∗^ | -0.0214^∗∗∗^ | -0.0191^∗∗∗^ |  |
| (0.00169) | (0.00213) | (0.00329) | (0.00393) | (0.00411) | (0.00377) | (0.00315) | (0.00340) | (0.00306) | (0.00327) |  |
| Control Mean / N .636 | .58 | .488 | .445 | .41 | .401 | .401 | .393 | .384 | .366 | 1,600,229 |

*Notes*: This table reports monthly estimates based on the regression model described by Equation (1) with no additional control variables across columns 1–10. Column 11 reports the monthly observation count for each subsample alongside control means in each month. Panel A reports estimates for patients aged 1–5, panel B reports estimates for patients aged 6–12, and panel C reports estimates for patients aged 13–17. Scaled estimates for *Y* 2020 are depicted in panel A of Figure 2. Standard errors are clustered at the state level. *, **, and ^∗∗∗^ denote 5%, 1%, and 0.1% significance levels, respectively.

1. **Robustness Checks and Additional Results**

We provide robustness checks and additional results using the IQVIA dataset and MEPS.

# Additional Core Results

In this part, we present the estimates associated with Figure 2 in table form. Tables [A4](#_bookmark2) and [A5](#_bookmark3) contain estimates by age group, specification (means, state FE, individual FE), and by month.

Table A5: Effect of Pandemic on Adherence: Variation By Age

| Mar | Apr | May | Jun | July | Aug | Sept | Oct | Nov | Dec | Obs |
| --- | --- | --- | --- | --- | --- | --- | --- | --- | --- | --- |
| (1) | (2) | (3) | (4) | (5) | (6) | (7) | (8) | (9) | (10) |  |
| Panel A: Within-Zip Code Estimates | | | | | | | | | | |
| 1–5 years old 0.0441^∗∗∗^ | 0.00774^∗^ | -0.0282^∗∗∗^ | -0.0386^∗∗∗^ | -0.0276^∗∗∗^ | -0.0295^∗∗∗^ | -0.0453^∗∗∗^ | -0.0527^∗∗∗^ | -0.0669^∗∗∗^ | -0.0655^∗∗∗^ | 1,418,334 |
| (0.00204) | (0.00302) | (0.00301) | (0.00224) | (0.00225) | (0.00219) | (0.00239) | (0.00261) | (0.00278) | (0.00339) |  |
| 6–12 years old 0.0382^∗∗∗^ | 0.0129^∗∗∗^ | -0.0188^∗∗∗^ | -0.0281^∗∗∗^ | -0.0182^∗∗∗^ | -0.0254^∗∗∗^ | -0.0415^∗∗∗^ | -0.0423^∗∗∗^ | -0.0484^∗∗∗^ | -0.0459^∗∗∗^ | 3,037,746 |
| (0.00200) | (0.00277) | (0.00262) | (0.00249) | (0.00328) | (0.00291) | (0.00307) | (0.00318) | (0.00290) | (0.00331) |  |
| 13–17 years old 0.0331^∗∗∗^ | 0.0227^∗∗∗^ | -0.00304 | -0.0115^∗∗^ | -0.00351 | -0.0103^∗∗^ | -0.0221^∗∗∗^ | -0.0233^∗∗∗^ | -0.0249^∗∗∗^ | -0.0210^∗∗∗^ | 1,599,289 |
| (0.00191) | (0.00249) | (0.00325) | (0.00390) | (0.00405) | (0.00374) | (0.00313) | (0.00341) | (0.00289) | (0.00331) |  |
| Panel B: Within-Individual Estimates | | | | | | | | | | |
| 1–5 years old 0.0496^∗∗∗^ | 0.00518 | -0.0416^∗∗∗^ | -0.0571^∗∗∗^ | -0.0486^∗∗∗^ | -0.0507^∗∗∗^ | -0.0795^∗∗∗^ | -0.100^∗∗∗^ | -0.128^∗∗∗^ | -0.136^∗∗∗^ | 529,349 |
| (0.00333) | (0.00486) | (0.00604) | (0.00450) | (0.00496) | (0.00604) | (0.00708) | (0.00449) | (0.00482) | (0.00501) |  |
| 6–12 years old 0.0353^∗∗∗^ | 0.00980^∗^ | -0.0277^∗∗∗^ | -0.0412^∗∗∗^ | -0.0334^∗∗∗^ | -0.0470^∗∗∗^ | -0.0722^∗∗∗^ | -0.0811^∗∗∗^ | -0.0963^∗∗∗^ | -0.0972^∗∗∗^ | 1,609,688 |
| (0.00322) | (0.00396) | (0.00404) | (0.00259) | (0.00376) | (0.00480) | (0.00529) | (0.00446) | (0.00463) | (0.00491) |  |
| 13–17 years old 0.0304^∗∗∗^ | 0.0185^∗∗∗^ | -0.0138^∗∗∗^ | -0.0263^∗∗∗^ | -0.0230^∗∗∗^ | -0.0380^∗∗∗^ | -0.0589^∗∗∗^ | -0.0671^∗∗∗^ | -0.0769^∗∗∗^ | -0.0763^∗∗∗^ | 750,848 |
| (0.00322) | (0.00326) | (0.00349) | (0.00303) | (0.00389) | (0.00458) | (0.00507) | (0.00421) | (0.00450) | (0.00427) |  |

*Notes*: This table reports the monthly effect of the pandemic on pediatric adherence based on the regression model described by Equation (1) across columns 1–10. Panel A reports the 2020 estimated effect for patients based on a within zip code specification, and panel B reports the 2020 estimated effect for patients based on a within individual specification. Scaled estimates for *Y* 2020 are depicted in panels B and C of Figure 2. Standard errors are clustered at the state level. ^∗^, ^∗∗^, and ^∗∗∗^ denote 5%, 1%, and 0.1% significance levels, respectively.

Figure A1: Robustness of Estimated Effect to Different Consumption Measures

(A) Baseline (B) Baseline


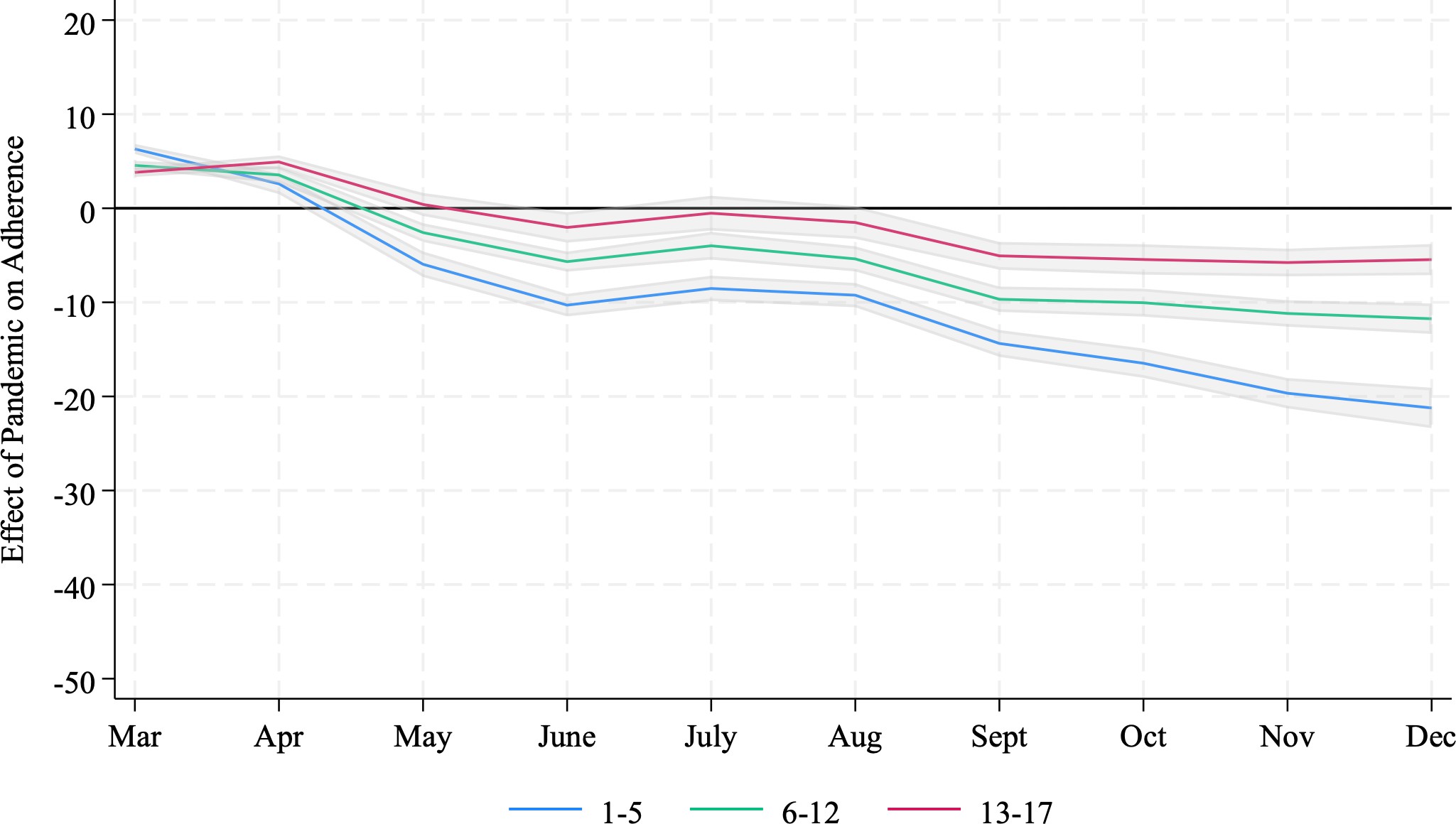

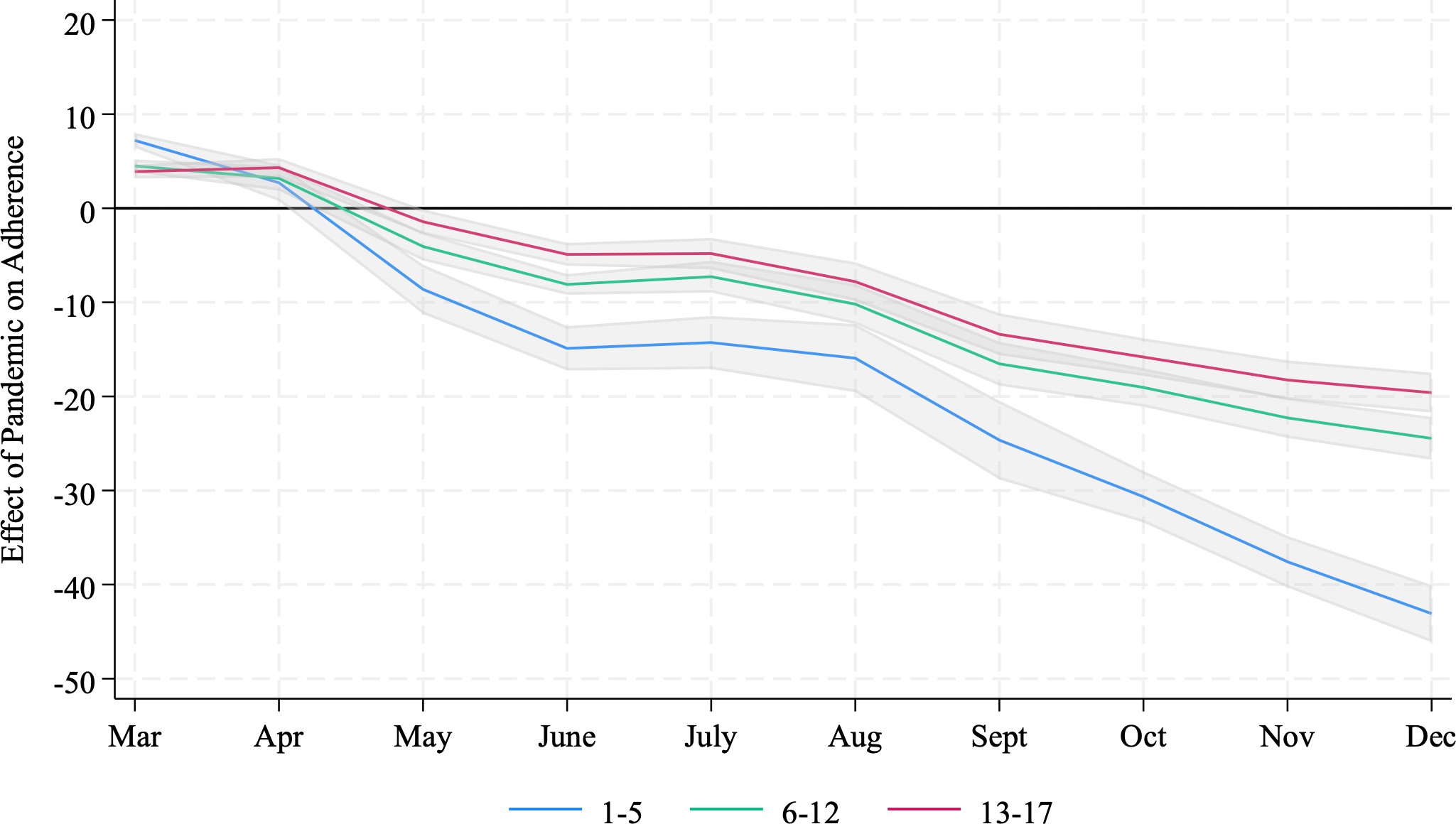


(C) *>*30% PDC (D) *>*30% PDC


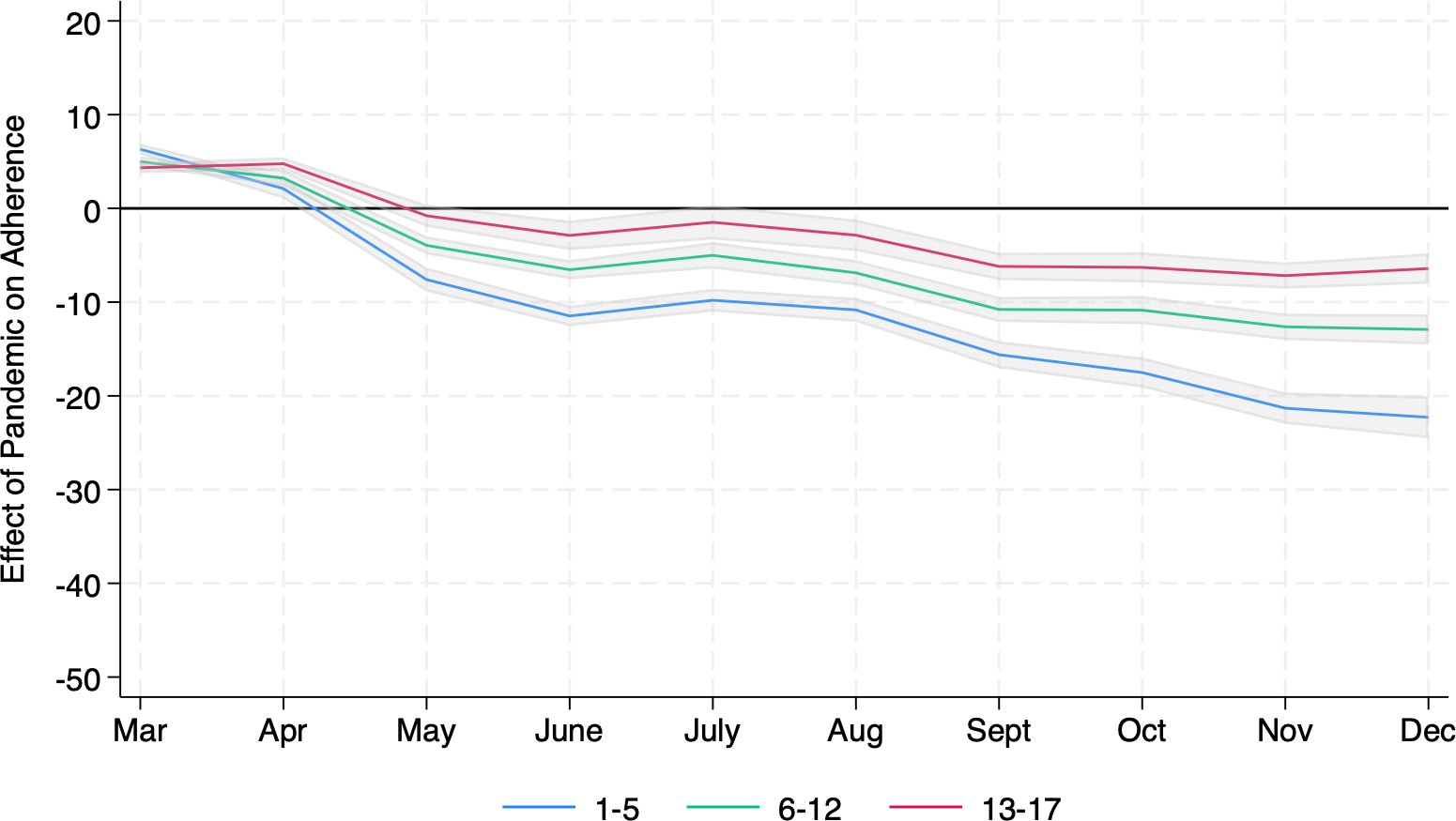

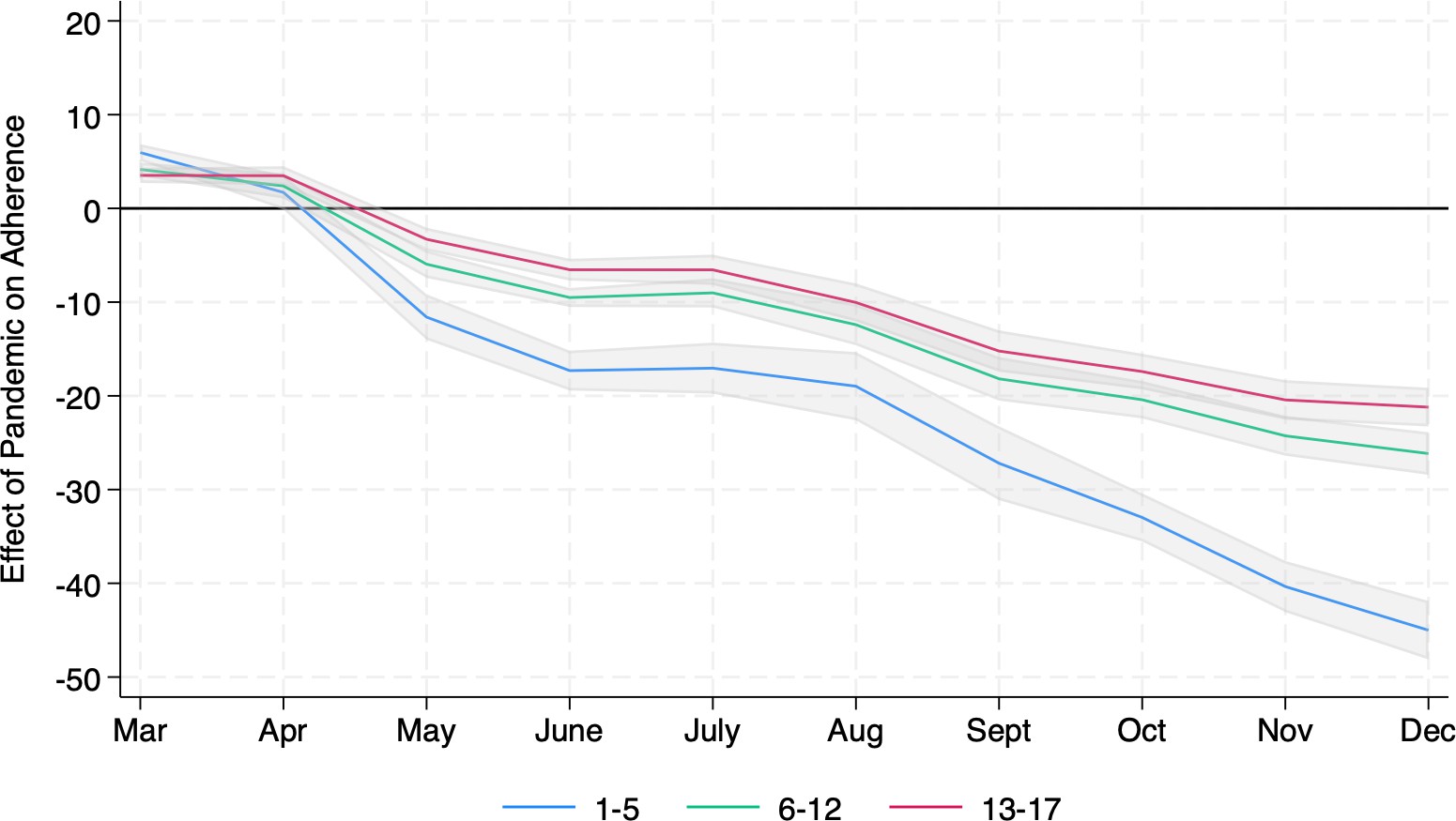


(E) *>*50% PDC (F) *>*50%


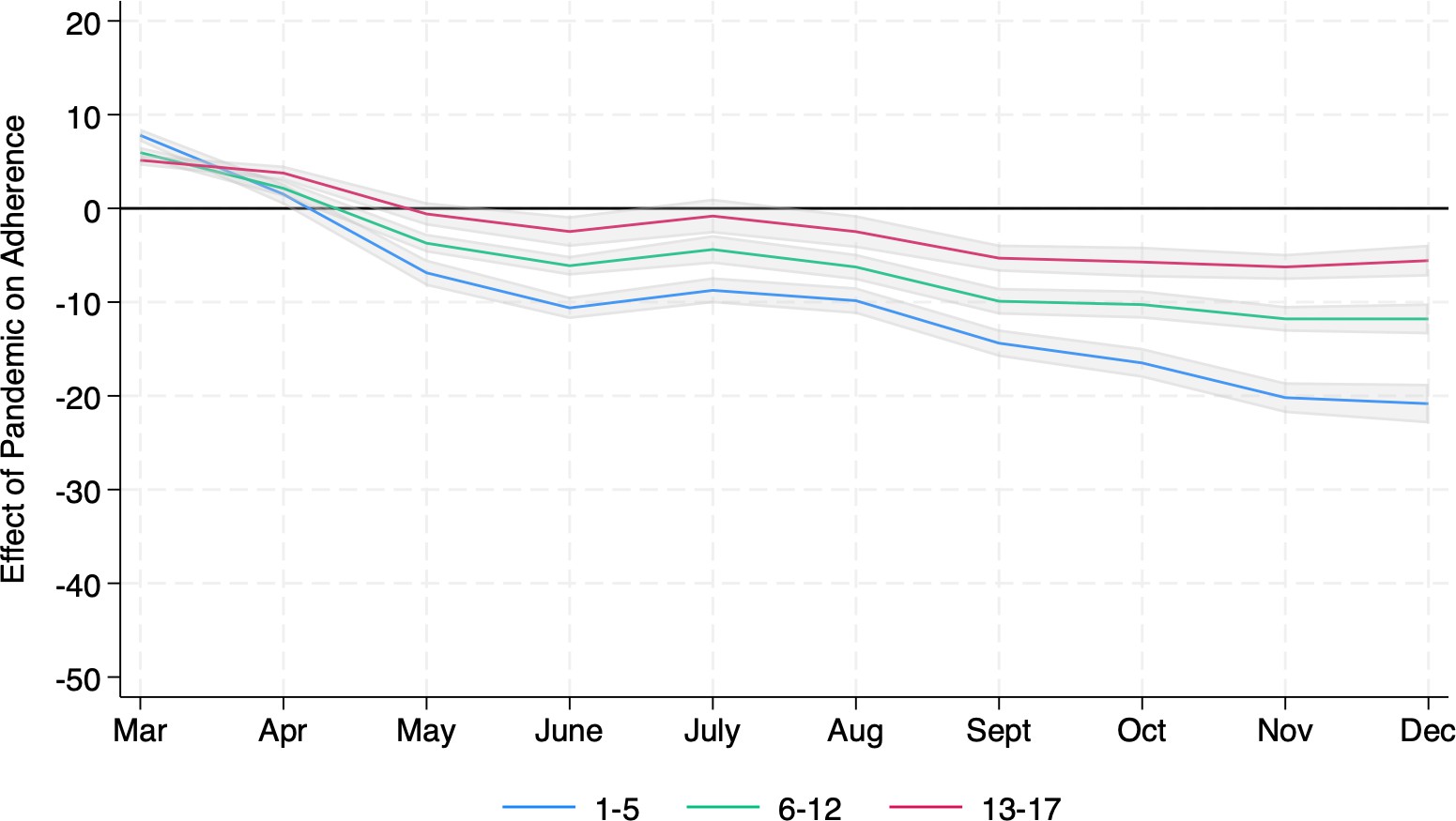

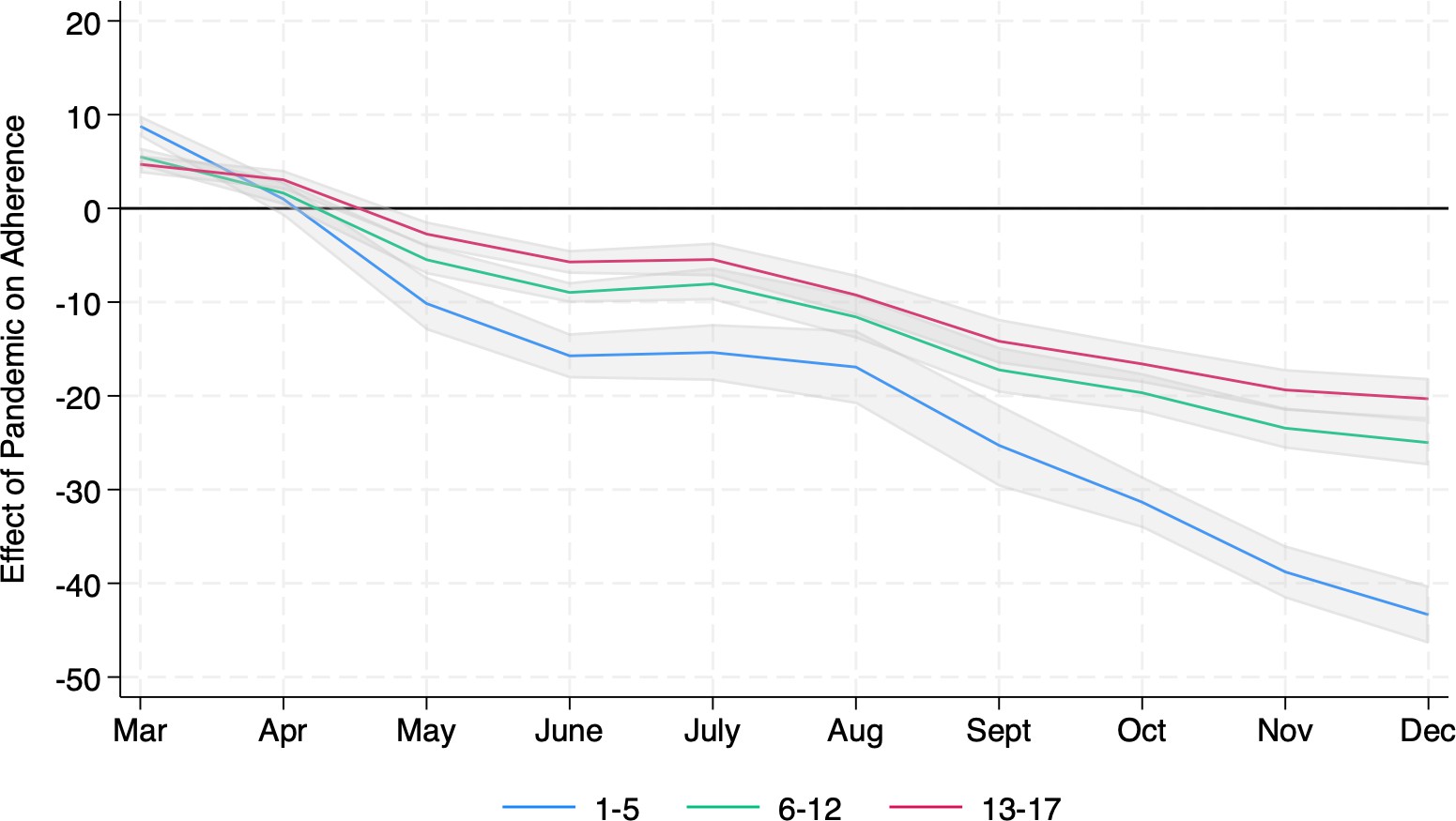


(G) MPR (H) MPR


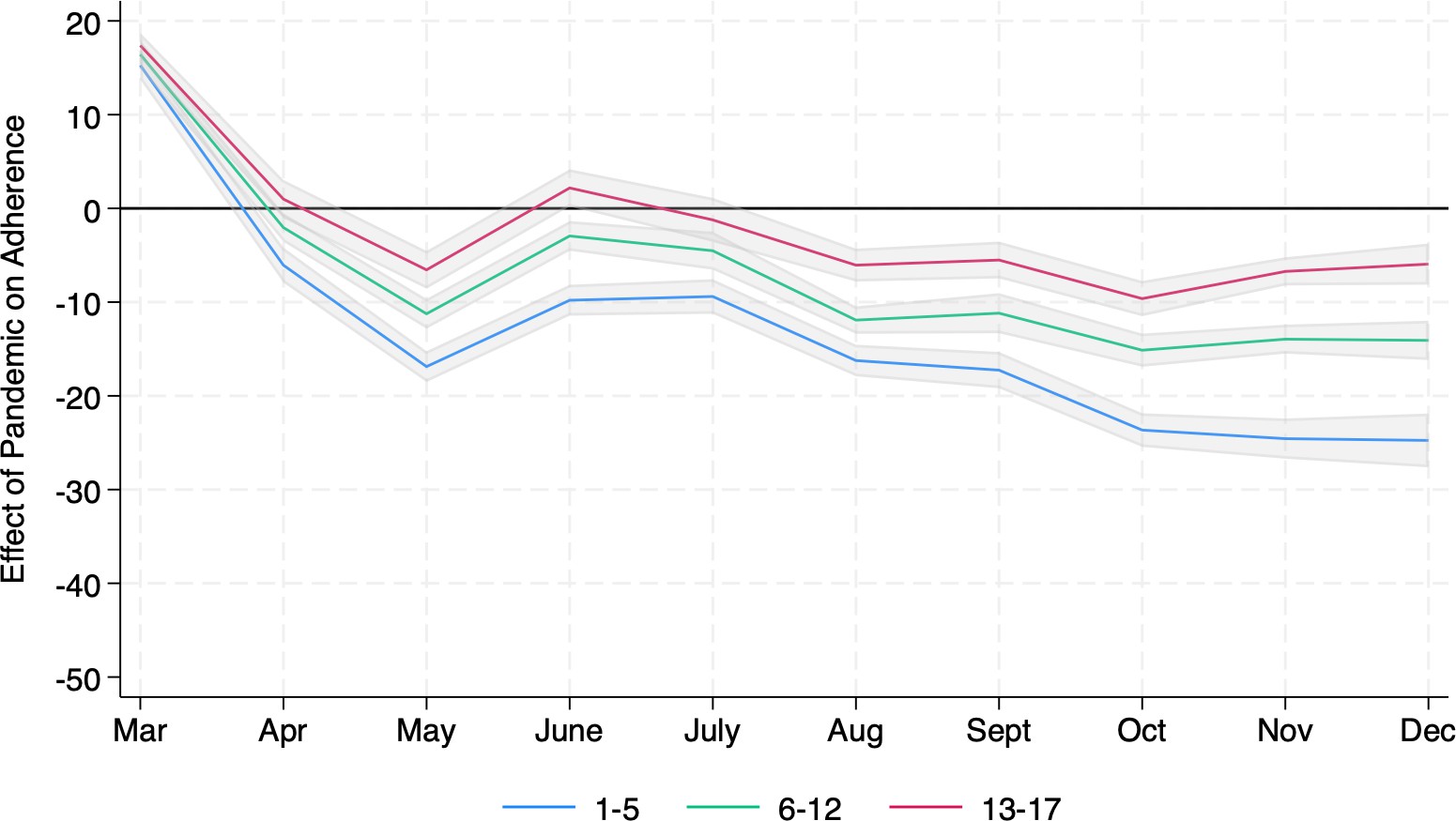

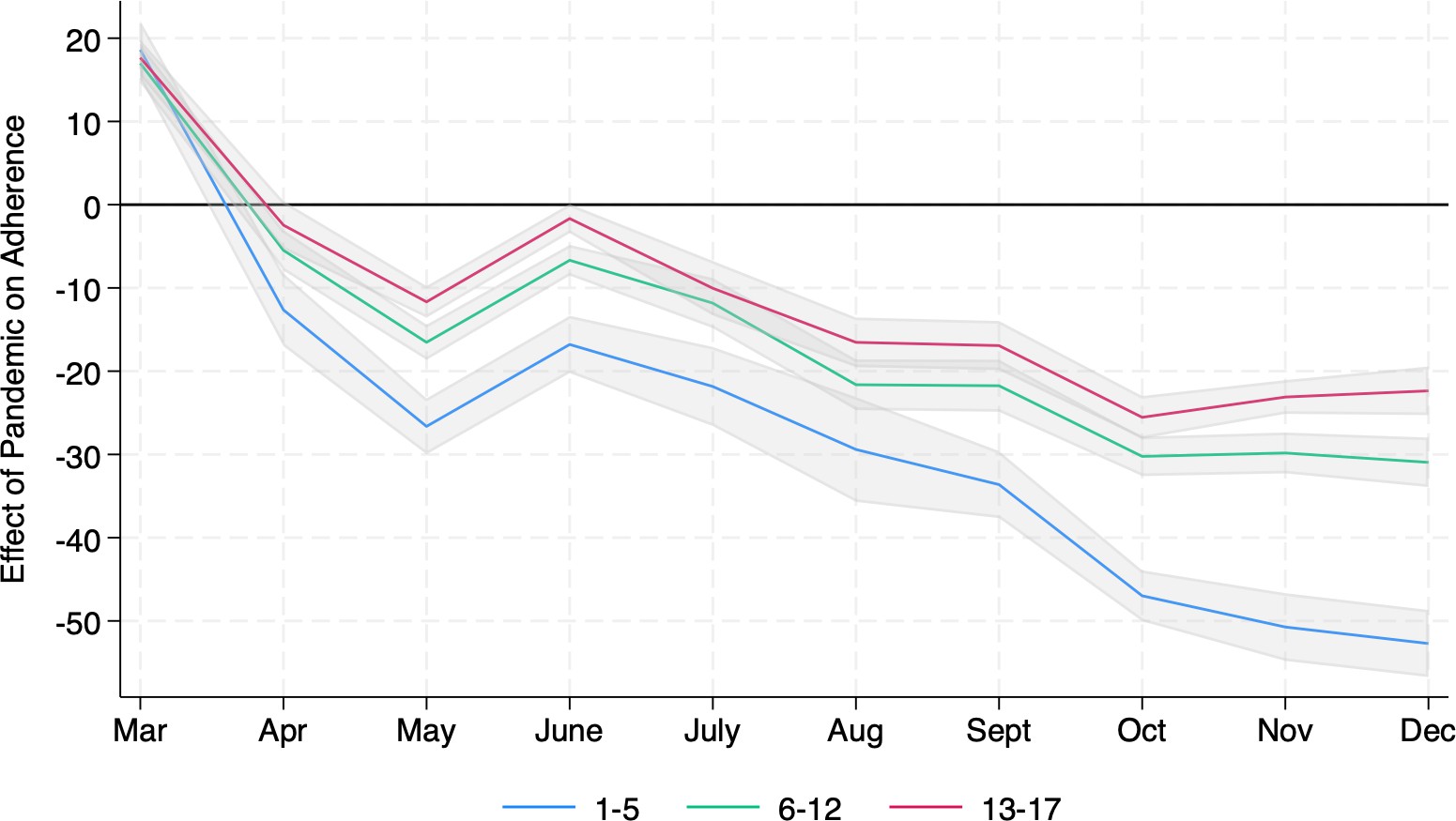


*Notes:* Panels (A) and (B) are repeated from Figure [2,](#_bookmark79) which is based on the raw proportion of days covered measure as the outcome. The remaining panels use an indicator for whether an individual had adherence above a given threshold in a given month (greater than 30 percent or greater than 50 percent) as the outcome or use an alternative Medical Possession Ratio (MPR) measure. Panels A, C, E, and F are based on a zipcode fixed effect specification and Panels B, D, F and H are based on an individual fixed effect specification. Although there are some differences across the three measures, we generally find quantitatively similar estimates.

# Additional Mechanisms Results, IQVIA

Next, we present additional results related to our mechanisms exploration in Section 4. First, Table [A6](#_bookmark4) presents point estimates associated with Figure [A3.](#_bookmark8) Next, we estimate non- parametric age responses at each age value and report estimates in Figure [A2.](#_bookmark5) Next, we report the adult version of the IQVIA horse race regressions in Table [A7.](#_bookmark6) Finally, we report summary stats for our pediatric diabetes sample in Table [A8](#_bookmark7)

42

Table A6: COVID Mechanisms

|  | Mar | Apr | May | Jun | July | Aug | Sept | Oct | Nov | Dec | Obs |
| --- | --- | --- | --- | --- | --- | --- | --- | --- | --- | --- | --- |
|  | (1) | (2) | (3) | (4) | (5) | (6) | (7) | (8) | (9) | (10) |  |
| Panel A: School Closure |  |  |  |  |  |  |  |  |  |  |  |
| Low | 0.0182^∗∗∗^ | -0.00977 | -0.0492^∗∗∗^ | -0.0610^∗∗∗^ | -0.0540^∗∗∗^ | -0.0707^∗∗∗^ | -0.0905^∗∗∗^ | -0.100^∗∗∗^ | -0.115^∗∗∗^ | -0.117^∗∗∗^ |  |
|  | (0.00342) | (0.00486) | (0.00597) | (0.00465) | (0.00852) | (0.0127) | (0.0146) | (0.0149) | (0.0139) | (0.0140) |  |
| Control Mean / N | .597 | .543 | .455 | .405 | .367 | .363 | .373 | .367 | .361 | .343 | 171,963 |
| High | 0.0345^∗∗∗^ | 0.0277^∗∗∗^ | -0.00907^∗∗^ | -0.0269^∗∗∗^ | -0.0203^∗∗∗^ | -0.0277^∗∗∗^ | -0.0557^∗∗∗^ | -0.0678^∗∗∗^ | -0.0797^∗∗∗^ | -0.0852^∗∗∗^ |  |
|  | (0.00239) | (0.00322) | (0.00297) | (0.00248) | (0.00356) | (0.00279) | (0.00407) | (0.00229) | (0.00251) | (0.00341) |  |
| Control Mean / N | .611 | .549 | .455 | .411 | .371 | .358 | .365 | .365 | .363 | .349 | 1,932,158 |
| Panel B: Air Quality |  |  |  |  |  |  |  |  |  |  |  |
| Low Improvement | 0.0284^∗∗∗^ | 0.0200^∗∗∗^ | -0.0194^∗∗∗^ | -0.0368^∗∗∗^ | -0.0334^∗∗∗^ | -0.0397^∗∗∗^ | -0.0629^∗∗∗^ | -0.0749^∗∗∗^ | -0.0877^∗∗∗^ | -0.0919^∗∗∗^ |  |
|  | (0.00436) | (0.00497) | (0.00508) | (0.00368) | (0.00359) | (0.00410) | (0.00535) | (0.00414) | (0.00340) | (0.00362) |  |
| Control Mean / N | .621 | .567 | .477 | .432 | .391 | .379 | .385 | .385 | .381 | .366 | 384,774 |
| High Improvement | 0.0313^∗∗∗^ | 0.0219^∗∗∗^ | -0.0151^∗∗∗^ | -0.0317^∗∗∗^ | -0.0259^∗∗∗^ | -0.0349^∗∗∗^ | -0.0612^∗∗∗^ | -0.0719^∗∗∗^ | -0.0842^∗∗∗^ | -0.0908^∗∗∗^ |  |
|  | (0.00267) | (0.00346) | (0.00321) | (0.00268) | (0.00337) | (0.00376) | (0.00472) | (0.00379) | (0.00398) | (0.00360) |  |
| Control Mean / N | .622 | .564 | .475 | .431 | .392 | .381 | .388 | .387 | .383 | .368 | 1,758,283 |
| Panel C: Telehealth |  |  |  |  |  |  |  |  |  |  |  |
| No Telehealth | 0.0304^∗∗∗^ | 0.0245^∗∗∗^ | -0.0137^∗^ | -0.0326^∗∗∗^ | -0.0283^∗∗∗^ | -0.0375^∗∗∗^ | -0.0616^∗∗∗^ | -0.0721^∗∗∗^ | -0.0856^∗∗∗^ | -0.0915^∗∗∗^ |  |
|  | (0.00328) | (0.00510) | (0.00581) | (0.00438) | (0.00639) | (0.00902) | (0.00993) | (0.0102) | (0.0104) | (0.0101) |  |
| Control Mean / N | .626 | .567 | .477 | .435 | .396 | .385 | .391 | .389 | .385 | .37 | 1,084,591 |
| Telehealth | 0.0305^∗∗∗^ | 0.0191^∗∗∗^ | -0.0190^∗∗∗^ | -0.0332^∗∗∗^ | -0.0260^∗∗∗^ | -0.0356^∗∗∗^ | -0.0612^∗∗∗^ | -0.0718^∗∗∗^ | -0.0840^∗∗∗^ | -0.0882^∗∗∗^ |  |
|  | (0.00280) | (0.00455) | (0.00455) | (0.00319) | (0.00388) | (0.00442) | (0.00534) | (0.00315) | (0.00331) | (0.00365) |  |
| Control Mean / N | .612 | .554 | .462 | .416 | .376 | .366 | .373 | .372 | .368 | .353 | 2,196,578 |
| Panel D: Delivery Channel | | | | | | | | | | | |
| Non-Mail | 0.0311^∗∗∗^ | 0.0205^∗∗∗^ | -0.0189^∗∗∗^ | -0.0345^∗∗∗^ | -0.0275^∗∗∗^ | -0.0368^∗∗∗^ | -0.0627^∗∗∗^ | -0.0731^∗∗∗^ | -0.0856^∗∗∗^ | -0.0902^∗∗∗^ |  |
|  | (0.00220) | (0.00360) | (0.00366) | (0.00256) | (0.00333) | (0.00419) | (0.00476) | (0.00392) | (0.00402) | (0.00410) |  |
| Control Mean / N | .609 | .55 | .458 | .414 | .375 | .365 | .372 | .371 | .368 | .352 | 3,235,801 |
| Mail | -0.00400 | 0.0121^∗∗^ | 0.0151^∗∗^ | -0.00631 | -0.0261^∗∗∗^ | -0.0338^∗∗∗^ | -0.0391^∗∗∗^ | -0.0497^∗∗∗^ | -0.0631^∗∗∗^ | -0.0718^∗∗∗^ |  |
|  | (0.00254) | (0.00427) | (0.00462) | (0.00447) | (0.00589) | (0.00626) | (0.00681) | (0.00545) | (0.00630) | (0.00608) |  |
| Control Mean / N | .917 | .91 | .827 | .754 | .714 | .692 | .665 | .65 | .638 | .622 | 69,514 |

*Notes*: This table reports monthly estimates of the response of pediatric monthly coverage to the pandemic across different subgroups based on a regression specification that includes individual fixed effects. Variable definitions provided in Table [A2](#_bookmark0) and [A3.](#_bookmark1) Standard errors are clustered at the state level. *, **, and ^∗∗∗^ denote 5%, 1%, and 0.1% significance levels, respectively.

Figure A2: Estimated Effect of COVID: Variation by Age

- - 1. March - December


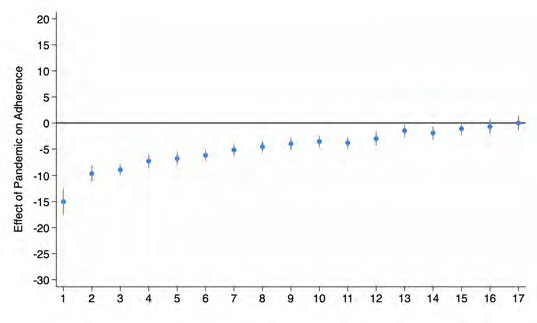


- - 1. March - July (c) August - December


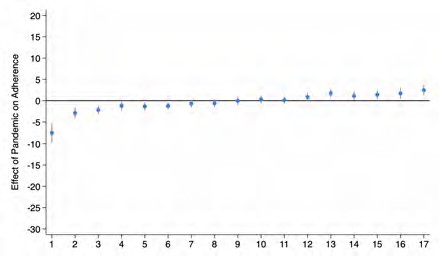

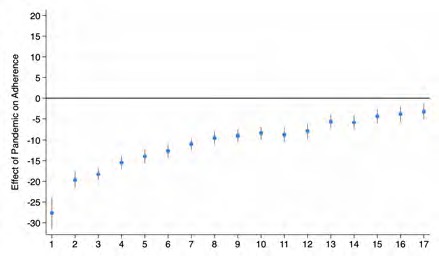


*Notes:* This figure plots the effect of the pandemic on drug adherence rates based on Equation (1), using provider zipcode fixed effects, and estimated separately for each age between 1 and 17. Panel (a) aggregates the effect in all post-pandemic months (March - December) and panels (b) and (c) aggregate the effect in two time periods (March - July and August - December) Point estimates are scaled by age-specific adherence rates observed during this same time period in 2019. 95% confidence intervals shown in shaded area.

Table A7: Collective Impact of Mechanisms for Adults: Horse Race

|  | Mar - Dec  (1) | Mar - July  (2) | Aug - Dec  (3) | Mar - Dec  (4) | Mar - July  (5) | Aug - Dec  (6) |
| --- | --- | --- | --- | --- | --- | --- |
| 2020 | 0.0269*** | 0.0368*** | 0.0171*** | 0.0256*** | 0.0340*** | 0.0173* |
|  | (0.00220) | (0.00194) | (0.00267) | (0.00599) | (0.00439) | (0.00808) |
| Age x 2020 |  |  |  | 0.000226*** (0.0000242) | 0.000276*** (0.0000236) | 0.000175*** (0.0000291) |
| Mail x 2020 |  |  |  | 0.0124*** (0.00190) | -0.00435* (0.00169) | 0.0291*** (0.00282) |
| AQI Improvement x 2020 |  |  |  | 0.00139 | 0.00162 | 0.00117 |
|  |  |  |  | (0.00148) | (0.00133) | (0.00182) |
| High Income x 2020 |  |  |  | -0.00624*** (0.00148) | -0.00346** (0.00119) | -0.00902*** (0.00183) |
| High Education x 2020 |  |  |  | 0.00246 | 0.00217 | 0.00274 |
|  |  |  |  | (0.00144) | (0.00129) | (0.00171) |
| White Collar x 2020 |  |  |  | -0.00395 | -0.000408 | -0.00750 |
|  |  |  |  | (0.00287) | (0.00204) | (0.00446) |
| High Minority x 2020 |  |  |  | -0.00169 | -0.00196 | -0.00142 |
|  |  |  |  | (0.00138) | (0.00124) | (0.00168) |
| Medicaid Payer x 2020 |  |  |  | 0.0183*** (0.00408) | 0.00840* (0.00356) | 0.0282*** (0.00483) |
| Medicaid Expansion x 2020 |  |  |  | -0.00530 | -0.00691** | -0.00369 |
|  |  |  |  | (0.00322) | (0.00236) | (0.00446) |
| Telehealth x 2020 |  |  |  | -0.00224 | -0.00331 | -0.00116 |
|  |  |  |  | (0.00365) | (0.00267) | (0.00508) |
| Urban x 2020 |  |  |  | 0.00201 | 0.00220 | 0.00182 |
|  |  |  |  | (0.00219) | (0.00153) | (0.00303) |
| High School Closure x 2020 |  |  |  | -0.00481* | -0.00283 | -0.00679** |
|  |  |  |  | (0.00190) | (0.00175) | (0.00231) |
| Control Mean | .572 | .629 | .516 | .572 | .629 | .516 |
| Zip Code FE | x | x | x | x | x | x |
| Observations | 128,001,280 | 64,000,640 | 64,000,640 | 128,001,280 | 64,000,640 | 64,000,640 |

*Notes*: Estimates are based on a regression model that compares adherence in 2020 to adherence in 2019 and 2018 for the adult population. Columns 1 and 3 report the average effect across all months, and columns 2, 3, 5, and 6 report estimates for a subset of months as indicated by column headers. Control means reflect the average pediatric adherence in the designated months in 2019. Variable definitions provided in Table [A2](#_bookmark0) and [A3.](#_bookmark1) All specifications include zip code fixed effects. Standard errors are clustered at the state level. *, **, and ^∗∗∗^ denote 5%, 1%, and 0.1% significance levels, respectively.

Table A8: Sample Statistics: Pediatric Diabetes Patients, January-March 2019

|  | Under 18  (1) | Under 6  (2) | 6 to 12  (3) | 13 to 18  (4) |
| --- | --- | --- | --- | --- |
| *Panel A: Individual Characteristics*  Adherence Rate | 0.80 | 0.83 | 0.82 | 0.79 |
| Patient Age | 12.65 | 4.21 | 9.68 | 15.25 |
| Share Female | 0.52 | 0.47 | 0.50 | 0.54 |
| Medicaid Payer | 0.12 | 0.06 | 0.11 | 0.13 |
| Third Party Payer | 0.76 | 0.64 | 0.73 | 0.79 |
| *Panel B: 2018 Local Geographic Characteristics* | | | | |
| Per-Capita Income, 2018 | 32,764 | 32,190 | 32,901 | 32,7 48 |
| Minority Share of Population, 2018 | 0.28 | 0.26 | 0.28 | 0.28 |
| Share Population with Some College, 2018 | 0.20 | 0.21 | 0.20 | 0.20 |
| Share of Population in Urban Area | 0.93 | 0.89 | 0.93 | 0.93 |
| Medicaid Expansion State | 0.61 | 0.60 | 0.62 | 0.61 |
| Observations | 177,899 | 11,674 | 59,797 | 106,428 |

*Notes:* This table provides summary statistics describing the pediatric diabetes users in 2019. Adherence statistics reflect prescriptions filled between January and March, and individual characteristics reflect the first prescription filled in 2019. Local geographic characteristics are matched based on the zip code of the provider associated with the first prescription filled. Variable definitions provided in Table [A2](#_bookmark0) and [A3.](#_bookmark1)

1. **Detailed Discussion of Additional Factors**

We discuss additional factors related to perceived need. Figure [A3](#_bookmark8) provides graphical confirmation that these channels have minimal quantitative impacts.

Figure A3: COVID-Specific Mechanisms and Effect on Adherence

(A) School Closures (B) Air Quality


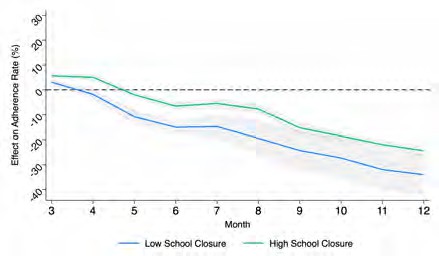

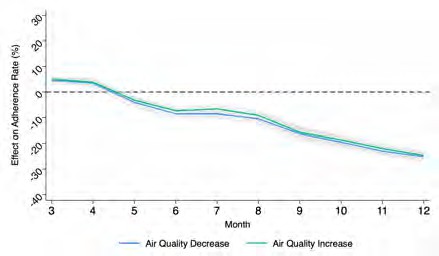


1. Telehealth


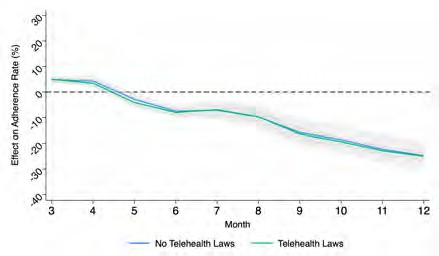


*Notes:* Panels (A) - (C) plot the effect of the pandemic on pediatric adherence as related to several COVID-specific changes. Panel (A) compares the effect for patients in counties with low and high school closure rates in Fall 2020. Panel (B) compares the effect based on whether Air Quality improved or declined between April and August 2020. Panel (C) compares the effect based on whether states require that public health insurance provide access to telehealth.

# School Closures

Nationwide lockdowns resulted in the sudden closure of schools to in-person learning. In-person schooling can affect pediatric adherence in several ways. For example, schools provide reminders to students to take their medication and reminders to parents to refill prescriptions (McClure et al., 2020). This mechanism would tend to reduce adherence in a remote learning environment, all else equal.

To investigate this conjecture, we use county-level data on in-person schooling from the U.S. School Closure and Distance Learning Database (Parolin and Lee, 2021) to create a county-level distribution of the share of schools with at least a 50 percent reduction in year-on-year attendance from September 2019 to September 2020. We categorize counties as high (low) school closure counties if they fall in the top (bottom) 25th percentile of this distribution (*High School Closure*). Figure [A3,](#_bookmark8) Panel (A) reports scaled estimates.

In short, we find a consistently small difference in responses between the two groups throughout the year. In particular, there is no additional difference in the fall, when there is more variation in in-person school attendance. Consistent with this, the estimated coefficient in the horse-race regression (Table 4) is quantitatively small (+1pp on a baseline of -10pp).

# Environmental Triggers

Next, we explore whether our results are driven by changes in medical need through reduced environmental triggers. Previous research notes that air quality had a major impact on health outcomes, particularly for older individuals (Finkelstein et al., 2023). In the case of COVID, stay-at-home orders effectively shut down commercial air travel and severely reduced road travel (Berman and Ebisu, 2020; Slezakova and Pereira, 2021; Venter et al., 2020), which may have led to even larger changes in air pollutant levels relative to a typical macroeconomic downturn. Because air pollution is an environmental trigger for the onset of asthmatic episodes, any associated improvements in air quality should serve to reduce medical need.

Figure [A3,](#_bookmark8) Panel (B) reports scaled estimates based on whether local air quality was unexpectedly better during the April to August 2020 period (*AQI Drop*).^[[1]](#footnote-1)^ We find very small differences across areas in terms of adherence responses. Our horse-race regression (Table 4, Models 4 and 6) shows that air quality improvement has minimal quantitative impacts on adherence to control medication. In unreported results, we do find an effect of air quality on prescriptions for as-needed medication, consistent with a reduced need for rescue inhalers due to better air quality.

# Access

The stay-at-home orders, along with reductions in person-to-person interactions, may have reduced access to in-person health care and, as a result, reduced access to prescriptions. However, previous research has documented that there was increased use of telehealth services during the pandemic (Volk et al., 2021). Thus, we test whether access plays a significant role by measuring the preparedness of individuals to switch to telehealth.

Using data from the Commonwealth Fund Issue Brief, we identify those states that had policies in place that required insurers to cover telehealth services (*Telehealth*) prior to 2020. The subset of patients using these services prior to the pandemic should have been the least affected by disruptions due to the closures of physician offices. Figure [A3,](#_bookmark8) Panel (C) reports scaled estimates. We find that responses are similar throughout the year, regardless of differential access to telehealth. The horse-race regression (Table 4) provides confirmation. We find a quantitatively negligible moderating effect.

# Insurance

To conclude our discussion, we briefly discuss the remaining factors included in Table 4. First, we note that variation in insurance plays a minor role in driving our results. Although prior literature has documented the importance of insurance coverage for adult adherence (Chandra et al., 2010; Finkelstein et al., 2012; Brot-Goldberg et al., 2017), children in the U.S. are much more likely to be insured due to public insurance programs. We compare the response of patients with fee-for-service Medicaid (*FFS Medicaid*) to all other payers. Consistent with this, we find that the mediating effect of Medicaid coverage was quantitatively small and statistically insignificant.^[[2]](#footnote-2)^

1. **Additional Results from MEPS and Marketscan**

# MEPS Data Construction and Summary Statistics

We discuss how we construct our MEPS asthma medication user panel, which we use to arrive at the estimates in Table 5.

To identify asthma-related scripts in MEPS, we rely on product names and national drug codes (NDCs) present in the IQVIA data. First, we take all product names and NDCs present in the IQVIA database on asthma scripts. Next, we match information on scripts in MEPS (Prescription Medicines files) to these two lists, keeping any scripts that match either the product name or the NDC. We then aggregate the data to a person-by-year level. Finally, we use the Full-Year Population Characteristics files to identify asthma medication users who continue to be surveyed in 2020, in order to assign zeros to individuals who are surveyed but do not report any asthma scripts. The data also record the age of the individual, allowing us to classify each individual by age. We use age in 2019 to classify individuals into groups, in order to keep the variable fixed over time.

Next, we use the Prescription Medicines files and Full-Year Population Characteristics files to construct parental measures. The Prescription Medicines files allow us to identify the total scripts of any kind filled by parents of kids taking asthma medication. The Population Characteristics file records education and also tracks self-reported mental health, insurance status, employment status, and hourly wage across the three survey rounds in a given year. This allows us to measure changes in insurance and employment status across rounds and also use 2019 self-reported mental health as a reference point for understanding an individual’s 2020 mental health status.

We also repeat the analysis for non-asthma chronic medication commonly taken by children under the age of 18 (Table 5, Panel B, Model 2). Specifically, we take all under-18 scripts in MEPS and select drugs that are taken by more than 10 children and have an average of three or more prescriptions per child. This set of drugs primarily contains allergy medication (*e.g.*, Zyrtec) and stimulants (*e.g.*, Adderall). We then repeat the same horse race analysis for the set of kids taking one of these non-asthma chronic medications in 2019. Table [A9](#_bookmark11) presents summary stats for asthma and non-asthma samples from 2020. The distribution of the outcome variable, days filled, motivates our usage of the Poisson regression. The summary statistics also motivate our usage of logs for parental prescriptions and hourly wage.

Table A9: MEPS Summary Statistics, Kids 2020

Variable Asthma Non-Asthma

|  | Median | Mean | S.D. |  | Median | Mean | S.D. |
| --- | --- | --- | --- | --- | --- | --- | --- |
| Days Filled (Q) | 0 | 78.95 | 214.81 |  | 30 | 269.74 | 572.61 |
| Parental Scripts | 10 | 18.48 | 25.16 |  | 10 | 23.23 | 32.71 |
| Mental Health | 2.33 | 2.34 | 0.85 |  | 2.33 | 2.34 | 0.83 |
| Lost Employment | 0 | 0.19 | 0.40 |  | 0 | 0.18 | 0.39 |
| Lost Insurance | 0 | 0.03 | 0.18 |  | 0 | 0.05 | 0.22 |
| Years Education | 14 | 13.74 | 2.43 |  | 14 | 13.69 | 2.53 |
| Hourly Wage | 17 | 24.89 | 26.20 |  | 17.45 | 25.71 | 25.88 |
| Individuals |  | 318 |  |  |  | 566 |  |

*Notes*: Summary statistics from 2020 for individuals under 18. “Days Filled” is the total days supplied across all prescriptions. The remaining variables are parental measures. “Parental Scripts” is the total prescriptions filled by parents. “Mental Health” is the average parental self-reported mental health status (1-5) across all rounds of the survey. Lost employment and lost insurance are measured based on changes across rounds in the survey, and equal one if there is any change to no employment or no insurance for one parent. “Years Education” is the maximum number of years of education across parents. Hourly wage is the total hourly wage across parents.

# MarketScan Data Construction and Results

Here, we use data from MarketScan to provide context on the relationship between pediatric prescription filling and their parents’ prescription filling. In the MEPS data, we find that parents filling prescriptions mitigates the negative response in children. However, MEPS does not have specific dates for prescription filling (and our IQVIA data does not contain links between parents and children).

To provide further context, we use data from MarketScan. MarketScan covers a large number of individuals who are insured by their employers. The data are not a random sample of the population but rather reflect the set of customers that use MarketScan services to better manage their health insurance. The advantage of the data is that it contains family identifiers and the dates on which scripts were filled. Our data covers the 1996 to 2013 period.

Our sample is the set of asthma prescriptions filled for children in 2013, the last year of our sample. We use the same set of products in our IQVIA data and find associated claims in MarketScan for children aged 17 or younger at the start of 2013. Then, for each prescription, we create indicators for the closest parental prescription fill. This includes parents filling prescriptions on the same day, within eight days, and in the same month/quarter/year.

Table [A10](#_bookmark12) presents the results. We observe 2,688,118 asthma prescriptions for 920,551 unique individuals under the age of 18. 14 percent of prescriptions are filled on the same day as a parent’s prescription. Half of the children’s asthma prescriptions come within 9 days of an adult prescription (19-day period). This co-occurrence rate increases to 62 percent, 79 percent, and 92 percent when counting prescriptions within the same month, quarter, and year, respectively. The results highlight the idea that pediatric prescriptions are sometimes, but not always, picked up at the same time as a parent picking up a prescription, leaving open the possibility that parental attention can have significant impacts on adherence rates.

Table A10: Parental Prescriptions Around Children’s Asthma Prescriptions

Same Day Within 9 days Same Month Same Quarter Same Year Rate 13.2% 50% 63.2% 79.8% 92.2%

*N* 920,551 individuals; 2,688,118 scripts

*Notes*: This table reports statistics on the timing of parental prescriptions relative to each pediatric asthma prescription. Source: 2013 MarketScan drug claims data.

# Trends in Hospitalizations and Emergency Room Visits

To provide evidence on trends in hospitalizations and emergency room visits, we again make use of MEPS data. We proceed as follows:

- - 1. For each year, we identify individuals with an asthma diagnosis in the “Medical Conditions” file. This corresponds to ICD-9 code 493 (before 2016) and ICD-10 code J45 (2016 and after).
    2. We then sort users into the same age bins as for the main analysis (1–5, 6–12, 13–17, 18+).
    3. We then count events from the “Hospital Inpatient Stays” and “Emergency Room Visits” files where the event is marked as related to a diagnosis in the Medical Conditions file. This is as granular a classification as available in MEPS due to privacy issues. Ideally, we would be able to confirm that the visit was for asthma and not other conditions.
    4. Finally, we calculate the average number of events within each age bin and year, weighting by the population weights provided by MEPS.

Figure [A4](#_bookmark14) shows the trend in the raw averages by group. Consistent with Binney et al. (2024), we find that there is generally a decreasing trend for pediatric asthma patients leading up to 2020, and also find a flat trend within adults. The number of events decreased sharply between 2019 and 2020 for three of the four groups, but only the youngest group exhibited a significant increase in 2021 and 2022.

We also formally compare outcomes at the individual level using the repeated cross-sections in MEPS. Formally, we run the following regression:

*yigt* = *αg* + *γt* + *β · Iage_i_<*6 + *γ · It*≥2021 *· Iage_i_<*6 + *ϵit* (D.1)

where *g* indexes the age group (under 6, 6–12, 13–17, and adults), *t* indexes the year, and the outcome of interest is total ER visits and inpatient stays for patient *i* in year *t*. We allow for year fixed effects (common trend across groups) and try to estimate the differential response of the youngest group in the two years after COVID.

Table [A11](#_bookmark15) reports the estimates. Column 1 uses the full population, and Column 2 uses just the pediatric population. We find statistically significant and quantitatively large and positive estimates for the differential impact on children under 6 (at the ten percent level in Column 1 and at the one percent level in Column 2). We caution that this is only circumstantial evidence using a small sample of nationally representative individuals.

Figure A4: Trends in Emergency Room Visits and Inpatient Stays by Age Group

.4

.3

ER Visits plus Hospital Stays

Under 6

.2 6 to 12

13 to 17

Adults

.1

0

2012 2014 2016 2018 2020 2022

Year

*Notes:* Trends in the average number of emergency room visits plus inpatient stays for individuals diagnosed with asthma within different age groups. We only include events that are marked as related to an existing medical condition.

Table A11: Differences in ER Visits and Hospitalizations between Youngest Group

ER Visits Plus Hospitalizations

|  | (1) | (2) |
| --- | --- | --- |
| Under 6 | -0.00310^∗^ | 0.00347^∗∗^ |
|  | (0.00166) | (0.00173) |
| Under 6 x Post 2021 | 0.0274^∗^ | 0.123^∗∗∗^ |
|  | (0.0153) | (0.0157) |
| Population | All | Under 18 |
| Observations | 217470 | 43600 |
| Adjusted *R*^2^ | 0.040 | 0.045 |

*Notes*: This table reports estimates of differences in hospitalization rates based on Equation [(D.1),](#_bookmark13) using MEPS data from 2013 to 2022. Regressions are weighted by population weights from MEPS. We report robust standard errors. ^∗^ *p <* 0*.*10, ^∗∗^ *p <* 0*.*05, ^∗∗∗^ *p <* 0*.*01.

1. Changes in air quality are measured by calculating the county-level change in air quality, as measured by the AQI, from April to August 2019 to 2020 relative to the change in air quality that occurred from 2018 to 2019. Lower AQI corresponds to better air quality. The binary variable “AQI Drop” captures whether a county had a negative difference (better air quality relative to trend). [↑](#footnote-ref-1)
2. We also find minimal differences between children in Medicaid expansion states versus other states. [↑](#footnote-ref-2)
